# Validation of diagnostic and predictive biomarkers for hereditary angioedema via plasma *N*‐glycomics

**DOI:** 10.1002/clt2.12090

**Published:** 2021-12-27

**Authors:** Zejian Zhang, Xue Wang, Jianqing Gu, Jianqiang Wu, Yang Cao, Yingyang Xu, Lisha Li, Kai Guan, Peng Liu, Jia Yin, Yuxiang Zhi, Shuyang Zhang

**Affiliations:** ^1^ Department of Medical Research Center State Key Laboratory of Complex Severe and Rare Diseases Peking Union Medical College Hospital Chinese Academy of Medical Sciences and Peking Union Medical College Beijing China; ^2^ Department of Allergy & Clinical Immunology National Clinical Research Center for Immunologic Diseases Peking Union Medical College Hospital Chinese Academy of Medical Sciences and Peking Union Medical College Beijing China; ^3^ Department of Cardiology State Key Laboratory of Complex Severe and Rare Diseases Peking Union Medical College Hospital Chinese Academy of Medical Sciences and Peking Union Medical College Beijing China

**Keywords:** biomarker, disease severity, galactosylation, plasma *N*‐glycome, sialylation

## Abstract

**Background:**

Hereditary angioedema (HAE) is a rare disease with heterogeneous clinical symptoms. It is vitally important to predict whether an HAE patient will develop severe symptoms in clinical practice, but there are currently no predictive biomarkers for HAE stratification. Plasma *N*‐glycomes are disease‐specific and have great potential for the discovery of non‐invasive biomarkers. In this study, we profiled the plasma *N*‐glycome of HAE patients from two independent cohorts to identify candidate biomarkers.

**Methods:**

Linkage‐specific sialylation derivatization combined with matrix‐assisted laser desorption/ionization time‐of‐flight mass spectrometry detection and automated data processing was employed to analyze the plasma *N*‐glycome of two independent type‐1 HAE cohorts.

**Results:**

HAE patients had abnormal glycan complexity, galactosylation, and α2,3‐ and α2,6‐linked sialylation compared to healthy controls (HC). The classification models based on dysregulated glycan traits could successfully discriminate between HAE and HC with area under the curves (AUCs) being greater than 0.9. Some of the aberrant glycans showed response to therapy. Moreover, we identified a series of glycan traits with strong associations with the occurrence of laryngeal or gastrointestinal angioedema or disease severity score. Predictive models based on these traits could be used to predict disease severity (AUC > 0.9). These results were replicated in an independent cohort.

**Conclusions:**

We reported the full plasma *N*‐glycomic signature of HAE for the first time, and identified potential biomarkers. These findings may play a critical role in predicting disease severity and guide the treatment of HAE in clinical practice. Further protein‐specific and prospective studies are needed to validate our findings.

## 1 INTRODUCTION

Hereditary angioedema (HAE) is a rare autosomal dominant disease that is primarily characterized by unpredictable and potentially life‐threatening subcutaneous or submucosal edema, which can affect the extremities, face, larynx, abdomen, or genitalia, with an incidence of 1/50,000 to 1/100,000.^1^, ^2^, ^3^ Typically, HAE is caused by C1 inhibitor (C1‐INH) deficiency (reduced level or abnormal function), and mutations in the SERPING1 gene.^1^, ^3^ HAE attacks can be spontaneous or provoked by emotional fluctuation, menstruation, fever, and trauma.^1^, ^2^ Currently, HAE is clinically diagnosed by combining family history, clinical symptoms, and laboratory tests (primarily complement tests).^1^ As HAE is a rare disease, and sometimes presents similar symptoms to other diseases, HAE patients are often misdiagnosed and treated incorrectly. For example, gastrointestinal angioedema episodes present clinical symptoms similar to those of acute intestinal obstruction, which often leads to unnecessary surgical intervention.^3^ Delaying diagnosis and treatment can have fatal consequences for HAE patients. Laryngeal angioedema attacks cause the obstruction of the upper airways, for which ineffective treatments (antihistamines, adrenaline, and corticosteroids) are often misused, and suffocation may ensue. However, specific target therapies, such as bradykinin B2 receptor antagonists, C1 inhibitor concentrate, kallikrein inhibitor, and sometimes tracheotomy, can prevent death in HAE patients.^4^ The situation is complicated by the heterogeneity of HAE clinical manifestations, even in individuals from the same family. Management strategies for different HAE patients differ greatly and may be adjusted based on the variable frequency and types of HAE attacks experienced by individual patients. However, there are currently no available methods to stratify HAE patients. Currently, danazol is the primary treatment option for short‐ and long‐term prophylaxis in China. However, danazol is associated with serious side effects, such as virilization, sequelae of obesity, and increased hepatic enzyme levels. Therefore, it is vital to develop tools used for patient stratification to guide individualized disease management.

N‐glycosylation is a common and functionally relevant post‐translational modification involving the synergistic action of multiple enzymes and transporters.^5^, ^6^, ^7^ Glycosylation is not template‐driven, but rather introduces variability in proteins that is independent of their corresponding DNA sequences.^7^ *N*‐glycans are highly diverse, and regulate multiple biological processes, from protein folding and stability to receptor‐ligand interactions and immune responses.^8^ Specific glycoforms depend on genetic, pathophysiological, and environmental factors.^9^ Plasma or serum protein *N*‐glycosylation in a given physiological state is stable, but can dramatically change in response to pathological conditions. Specific glycosylation features of the plasma *N*‐glycome are related to various pathological conditions, such as Down syndrome,^10^ rheumatoid arthritis,^11^ inflammatory bowel disease,^12^ and cancers.^13^, ^14^ Importantly, the plasma *N*‐glycome is often disease‐specific. The plasma *N*‐glycome may represent a non‐invasive biomarker, and provide a more thorough understanding of specific disease mechanisms. Currently, little is known regarding *N*‐glycosylation in HAE.

In this study, we investigated the plasma protein *N*‐glycomic features in HAE patients, HAE patients after treatment, and healthy controls (HCs). Thereafter, we validated our findings in an independent cohort. As the function of sialylation depends on the linkage type, we employed the linkage‐specific sialic acid derivatization method developed by Reiding et al.^15^ to distinguish between the α2,3‐ and α2,6‐linked sialic acids on the non‐reducing end of plasma glycans. We used matrix‐assisted laser desorption/ionization time‐of‐flight (TOF) mass spectrometry (MALDI‐TOF MS) to profile the *N*‐glycome of the cohort.^15^ Our primary objective was to reveal the plasma *N*‐glycomic features that are HAE‐specific, and to discover and validate putative glycan biomarkers for HAE diagnosis, stratification, and monitoring.

## 2 METHODS

### 2.1 Study design and participants

In the present study, the discovery cohort enrolled 21 drug‐naïve type‐1 HAE patients, 12 type‐1 HAE patients after treatment, and 50 HCs. The independent validation cohort consisted of 18 drug‐naïve type‐1 HAE patients, 30 type‐1 HAE patients after treatment, and 40 HCs. The plasma of each individual from the two cohorts was collected from the Peking Union Medical College Hospital (Beijing, China). The age and sex of the three subgroups in each cohort were matched where possible. Plasma samples were stored at −80°C. HAE patients were diagnosed on the basis of medical history, clinical symptoms, and clinical parameters from the laboratory: (1) a history of recurrent episodes of subcutaneous and/or submucosal edema (such as edema affecting the extremities, face, larynx, abdomen, or genitalia) and (2) confirmed lower levels of clinical parameters, including C4 and C1‐INH antigen. This study was approved by the regional ethics committee of Peking Union Medical College Hospital (No. HS‐2402). All study participants gave written informed consent.

### 2.2 Plasma *N*‐glycome detection and MS data (pre‐)processing

Plasma samples were enzymatically treated to release *N*‐glycans according to a previously reported method.^15^ Briefly, 10 μL of 2% SDS was added to 5 μL of plasma, and the mixture was incubated for 15 min at 65°C. The *N*‐glycans were then released by the addition of 10 μL of release mixture (1 U PNGase F, 2% NP‐40, and 2.5 × PBS), followed by overnight incubation at 37°C. To derivatize sialic acids, α2,3‐linked sialic acids were lactonized, and α2,6‐linked sialic acids were ethyl‐esterified, allowing mass‐based distinction between the sialic‐acid linkage variants.^15^ Released *N*‐glycans were then enriched by hydrophilic interaction liquid chromatography solid‐phase extraction (HILIC‐SPE) micro‐tips using cotton thread as the stationary phase packed in the pipette tips and enriched glycans were eluted with Milli‐Q water according to the method described previously.^13^, ^14^ HILIC‐SPE cotton‐tips allowed the removal of salts, most deglycosylated peptides, and detergents from glycoconjugate samples. In addition, subsequent MALDI‐TOF MS glycan profiles were very repeatable with different tips.^15^ The sialylated *N*‐glycans were detected simultaneously with non‐sialylated *N*‐glycans using MALDI‐TOF MS in positive‐ion mode as previously described, with minor modification.^14^ Briefly, 1 μL of purified glycans was mixed with 1 μL of matrix (5 mg/mL super‐2,5‐dihydroxybenzoic acid with 1 mM NaOH in 50% acetonitrile) on the target plate. Mass spectra were obtained using a rapifleXtreme MALDI‐TOF MS (Bruker Daltonics). The instrument was equipped with a Smartbeam‐3D laser and was controlled using flexControl 4.0 (Bruker Daltonics). The laser was fired 5000 times per spot in a random walking pattern at a frequency of 5000 Hz. The mass range was set to 1000–5000 m/z. The instrument was calibrated using external calibrants (Bruker Peptide Calibration Standard II).

Raw MS data was baseline‐subtracted and smoothed. The MS data was transformed to .xy files and re‐calibrated using selected glycan signals as calibrants (Table S1), using the in‐house developed software by Jansen et al. (MassyTools, version 0.1.8.1.2).^16^ Thereafter, summed spectra were generated for each biological group (untreated HAE, treated HAE, and HC) and the quality control group (randomly distributed plasma standard). For each summed spectrum, mono‐isotopic peaks with good signal to noise (S/N; >3), good relative intensity (>0.1%), and good isotopic patterns were filtered for further analysis, and 90 mono‐isotopic peaks were assigned to *N*‐glycan structures using Glycoworkbench^17^ as well as previously confirmed glycan compositions.^15^ Finally, an *N*‐glycan composition list was generated for use in subsequent targeted data extraction. The peak intensities of the putative *N*‐glycans were extracted as peak area (background‐corrected) for all samples using the *N*‐glycan composition list and MassyTools. Further processing of the extracted data was done in Microsoft Excel. Glycan structures were excluded after applying cut‐offs for S/N (>9), mass accuracy (ppm error < |20|), isotopic pattern quality (QC score < 25%), and the minimum percentage (>50%) of presence in all spectra of HAE, HC, or quality control plasma samples.^14^ After curation, 59/90 *N*‐glycans (Table S2) were included for quantitative analysis. The sum of the areas of these remaining 59 compositions per spectrum was normalized to one. To combine the exact effects of individual glycans sharing similar glycan structures, and to enable interpretation of their biological effects, 82 derived *N*‐glycan traits were calculated from the directly detected *N*‐glycan traits based on their common structural characteristics (Table S3). The formulas used for the calculation of the derived glycan traits are given in Table S3, and calculations were performed in RStudio. The subject of the calculation is represented by the last letter, for example, sialylation (S), and the group on which it is calculated by the preceding letters, for example, triantennary fucosylated species (A3F). This, for instance, translates A3FS into the sialylation within triantennary fucosylated species.^14^, ^15^, ^18^ Differential derived *N*‐glycan traits demonstrate that changes to glycosylation are shared by a series of structurally related *N*‐glycans.^14^

### 2.3 Statistical analysis

Comparisons were made for derived *N*‐glycan traits between two subgroups in each cohort (untreated HAE vs. HC, treated HAE vs. HC) using the Mann‐Whitney‐Wilcoxon test (because the data was non‐normally distributed). Multiple testing correction was performed to adjust the significance threshold (*p* = 0.05/82). Regression analysis was performed in RStudio. The diagnostic/predictive potential of the individual *N*‐glycan traits was further evaluated based on receiver operator characteristics (ROC). Classification/prediction biomarker models were constructed using multivariate algorithms for support vector machines (SVMs) based on the differentially expressed derived *N*‐glycan traits or glycan traits strongly associated with clinical symptoms. ROC curves were obtained by Monte Carlo cross validation (MCCV). In each MCCV, two‐thirds of the samples were used to assess the importance of each glycan trait, and the remaining one‐third was used to validate the biomarker models generated in the first step. The top‐ranking important glycan traits were subsequently used to construct predictive biomarker models. These steps were repeated, and the performance of each model was calculated and compared. The area under the curve (AUC) of ROC curves and predictive accuracy were used to assess the performance of the output models. We considered AUCs ≥ 0.9 to represent highly accurate tests, whereas 0.8 ≤ AUCs < 0.9 represented accurate tests, and 0.7 ≤ AUCs < 0.8 represented moderately accurate tests.

## 3 RESULTS

**FIGURE 1:**
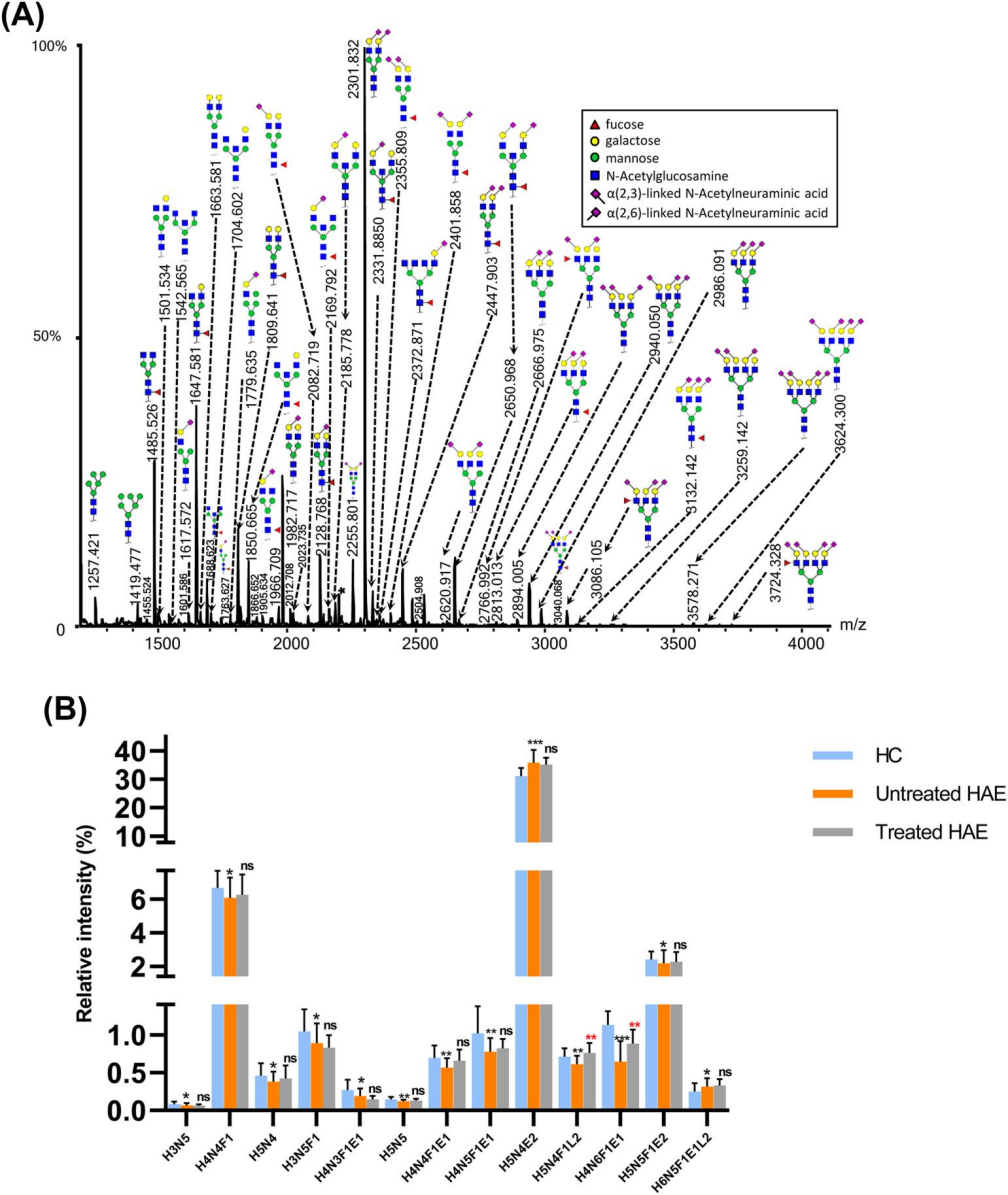
(A) A typical matrix‐assisted laser desorption/ionization time‐of‐flight mass spectrometry (MALDI‐TOF‐MS) spectrum of the plasma protein *N*‐glycome in hereditary angioedema patients. Spectra were recorded in positive‐ion reflectron mode on a Bruker rapifleXtreme mass spectrometer. Major *N*‐glycan peaks were annotated and assigned to compositions. The presence of structural isomers cannot be excluded. Asterisks (*) indicate by‐products. (B) The bar plot with standard deviation bars for the directly detected glycan traits changing in untreated hereditary angioedema (HAE) patients compared to that in healthy controls of the cohort. ***: *p*‐value < 0.001, **: *p*‐value < 0.01, *: *p*‐value < 0.05, ns: not significant. The “*” or “ns” on the top of the histogram of the treated HAE represent the statistical significance for “HC versus untreated HAE,” on the top of treated HAE represent the statistical significance for “untreated HAE versus treated HAE.” HAE, hereditary angioedema; HC, healthy control; H, hexose; N, N‐acetylhexosamine; F, deoxyhexose (fucose); L, actonized N‐acetylneuraminic acid (α2,3‐linked); E, ethyl esterified N‐acetylneuraminic acid (α2,6‐linked)

The clinical characteristics of two cohorts are presented in Table 1. Fifty‐nine directly detected *N*‐glycan compositions passed our quality criteria for subsequent quantification. Eighty‐two derived *N*‐glycan traits were generated based on the structural features of the directly detected glycans. Features of the derived *N*‐glycan traits include the number of antennae (A), fucosylation (F), bisection (B), galactosylation (G), α2,3‐linked (L), and α2,6‐linked sialylation (E) (Figure 1; Tables S2–S3). The consistent quality and overall method repeatability of the data was assessed by including plasma standards (Table S4). The average RSD of the directly detected *N*‐glycan traits (top 20) and all 82 derived traits was 3.93% and 2.32%, respectively (Table S4). Derived traits appear to have better technical robustness than directly detected glycans. When doing analysis, we found 13 directly detected glycan traits were significantly changed in HC compared with untreated HAE (Figure 1B). As derived *N*‐glycan traits could combine the exact effects of individual glycans sharing similar glycan structures, facilitate interpretation of biological effects, and have higher repeatability than the individual glycan traits from which they were calculated,^19^ we subsequently focused on the derived *N*‐glycan traits for the comparison and analysis in the present study.

**TABLE 1:** Clinical characteristics of all participants arranged by subgroups

	Discovery cohort			Validation cohort		
	Untreated HAE	Treated HAE	HC	Untreated HAE	Treated HAE	HC
Sample size	*n* = 21	*n* = 12	*n* = 50	*n* = 18	*n* = 30	*n* = 40
Age (years), mean (SD)	33.1 (11.0)	41.5 (13.4)	35.6 (7.7)	43.3 (11.9)	42.6 (12.9)	42.5 (9.9)
Gender, male (%)	10 (47.6%)	4 (33.3%)	25 (50.0%)	5 (27.8%)	9 (30.0%)	12 (30.0%)
Percentage of patients who have experienced laryngeal edema (%)	84.2%	75.0%	NA	55.6%	73.3%	NA
Percentage for patients who have experienced abdominal pain (%)	68.4%	66.7%	NA	77.8%	76.7%	NA
No. with HAE severity score (0 point)	0	0	NA	0	8	NA
No. with HAE severity score (1 point)	0	0	NA	2	1	NA
No. with HAE severity score (2 points)	2	2	NA	2	2	NA
No. with HAE severity score (3 points)	2	1	NA	1	7	NA
No. with HAE severity score (4 points)	9	5	NA	5	2	NA
No. with HAE severity score (5 points)	5	3	NA	6	5	NA
No. with HAE severity score (6 points)	0	0	NA	1	1	NA
No. with HAE with severity score (NA)	3	1	NA	1	4	NA
C4 (g/l), normal reference value: 0.100–0.400 g/l	0.078 (0.056–0.129)	0.098 (0.062–0.159)	NA	0.032 (0.014–0.066)	0.062 (0.052–0.095)	NA
CI‐INH (g/l), normal reference value: 0.21–0.39 g/l	0.080 (0.050–0.110)	0.075 (0.058–0.123)	NA	0.050 (0.040–0.060)	0.060 (0.055–0.095)	NA

*Note*: Clinical parameters are represented as median and interquartile range. The clinical severity score (0–7 points) system was performed as follow: frequency of attacks >1/month, 1’: skin edema of any area ever, 1’: single submucous site involvement, 1’: multiple submucous sites involvement, 2’; emergency visit ever, 1’; improper abdominal operation ever, 1’; tracheotomy, 1’.

Abbreviations: HAE, hereditary angioedema; HC, healthy control; NA, not available.

### 3.1 Identification of plasma *N*‐glycomic features in HAE patients

**FIGURE 2:**
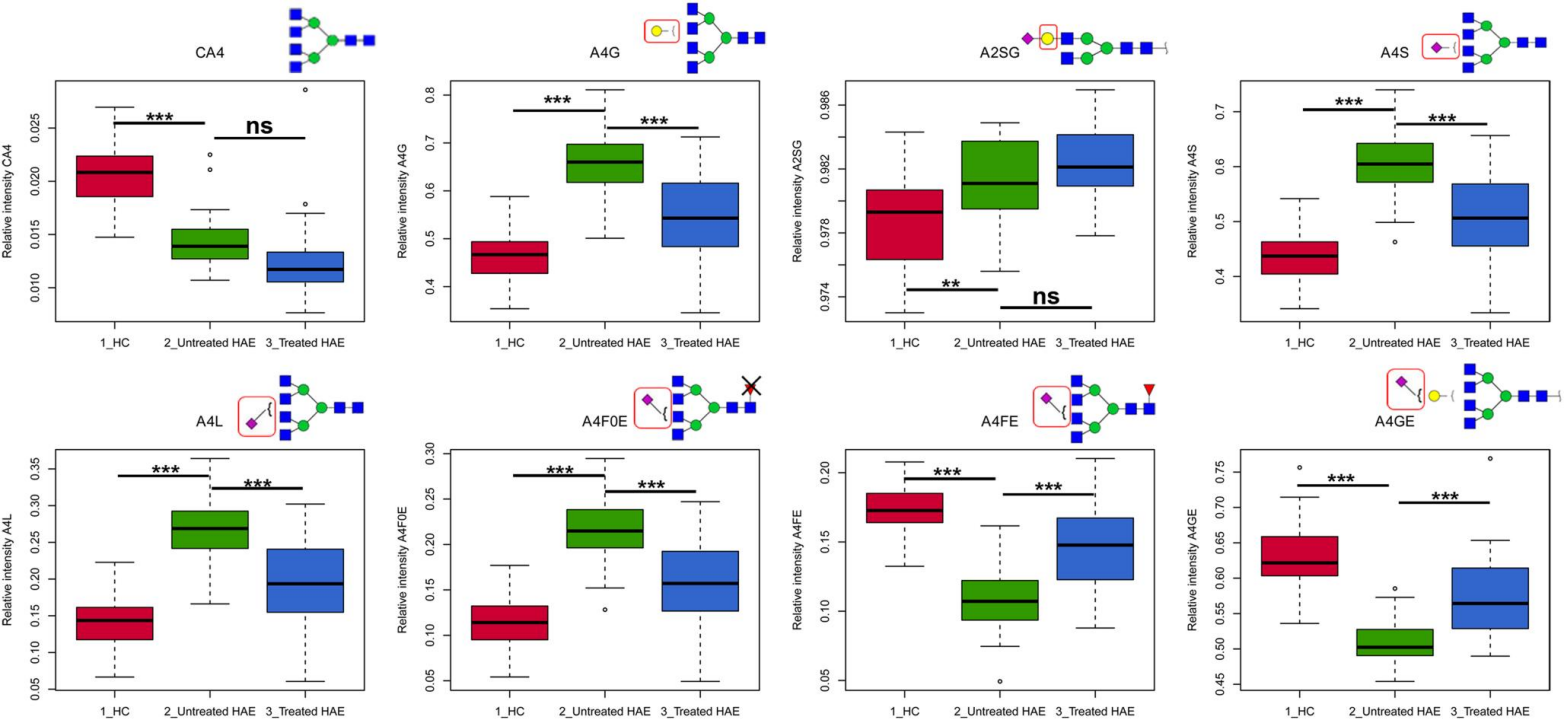
Boxplots of derived glycan traits changing in untreated hereditary angioedema (HAE) patients compared to that in healthy controls in the validation cohort. The boxplot for the group of HAE patients after receiving treatment is also shown. ***: *p*‐value < 0.001, **: *p*‐value < 0.01, *: *p*‐value < 0.05, ns: not significant (after multiple testing correction). HAE, hereditary angioedema; HC, healthy control

Eight replicated derived *N*‐glycan traits differed significantly between patients with drug‐naïve type 1 HAE and HCs in the two independent cohorts (Figure 2; Table 2; Figure S1). *N*‐glycomes in HAE patients had different antennarity within the complex‐type glycans (CA) than in HCs. The level of tetra‐antennary glycans within complex type (CA4) in HAE patients decreased compared with that in HCs (Figure 2; Table 2; Figure S1). The levels of galactosylation of tetra‐antennary glycans (A4G) and galactosylation of sialylated diantennary glycans (A2SG) in HAE patients were higher than that in HCs (Figure 2; Table 2; Figure S1). Patterns of sialylation also differed between HAE patients and HCs. The level of sialylation of tetra‐antennary glycans (A4S) was higher in subjects with HAE than in HCs, which was primarily caused by the increase in α2,3‐linked sialylation of tetra‐antennary glycans (A4L; Figure 2; Table 2; Figure S1). Increased α2,6‐linked sialylation of non‐fucosylated tetra‐antennary glycans (A4F0E) was also observed in HAE (Figure 2; Table 2; Figure S1). In contrast, α2,6‐linked sialylation of fucosylated tetra‐antennary glycans (A4FE) and α2,6‐linked sialylation per galactose of tetra‐antennary glycans (A4GE) decreased in HAE patients compared to that in HCs (Figure 2; Table 2; Figure S1). No differences were found for fucosylation (F), bisection (B), hybrid (THy), and high‐mannose structures (TM) between HAE patients and HCs.

**TABLE 2:** The dysregulated derived *N*‐glycan traits in hereditary angioedema (HAE) patients compared to healthy controls in the discovery and validation cohort

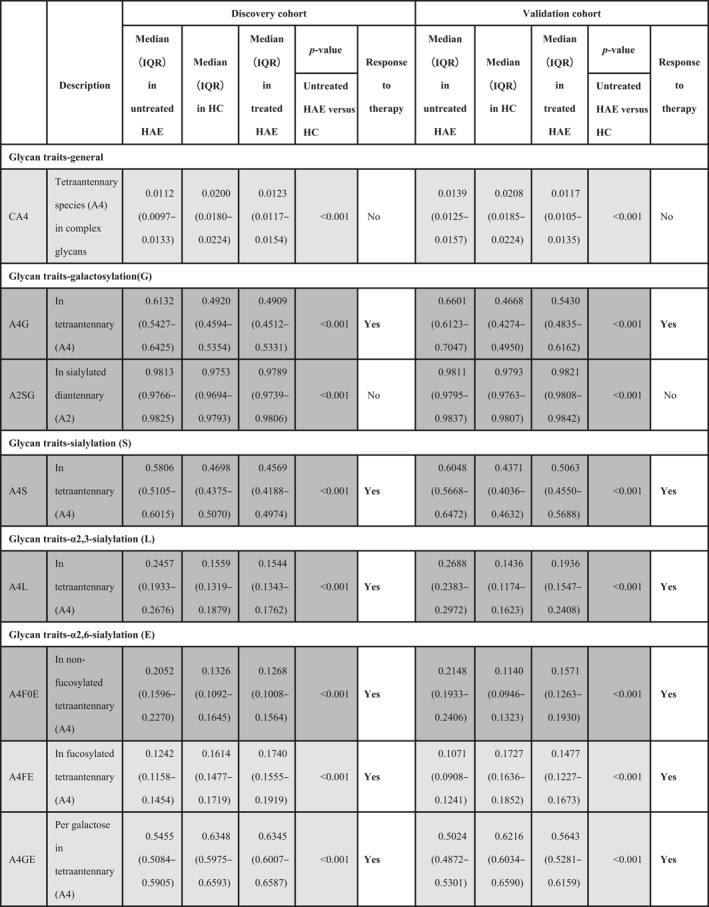

*Note*: Table showing the descriptions of the derived traits, median values (IQR), *p*‐values (Mann‐Whitney‐Wilcoxon test), and the response to therapy. The significance threshold was corrected for multiple tests. Differences were considered significant if *p* < 6.10E‐4 (*p*‐value of 0.05/82). Dark and light gray shading indicate up‐ and down‐regulation in untreated HAE compared with in HCs, respectively.

Abbreviations: HAE, hereditary angioedema; HC, healthy control.

### 3.2 Performance of plasma glycan traits for diagnosing HAE

**FIGURE 3:**
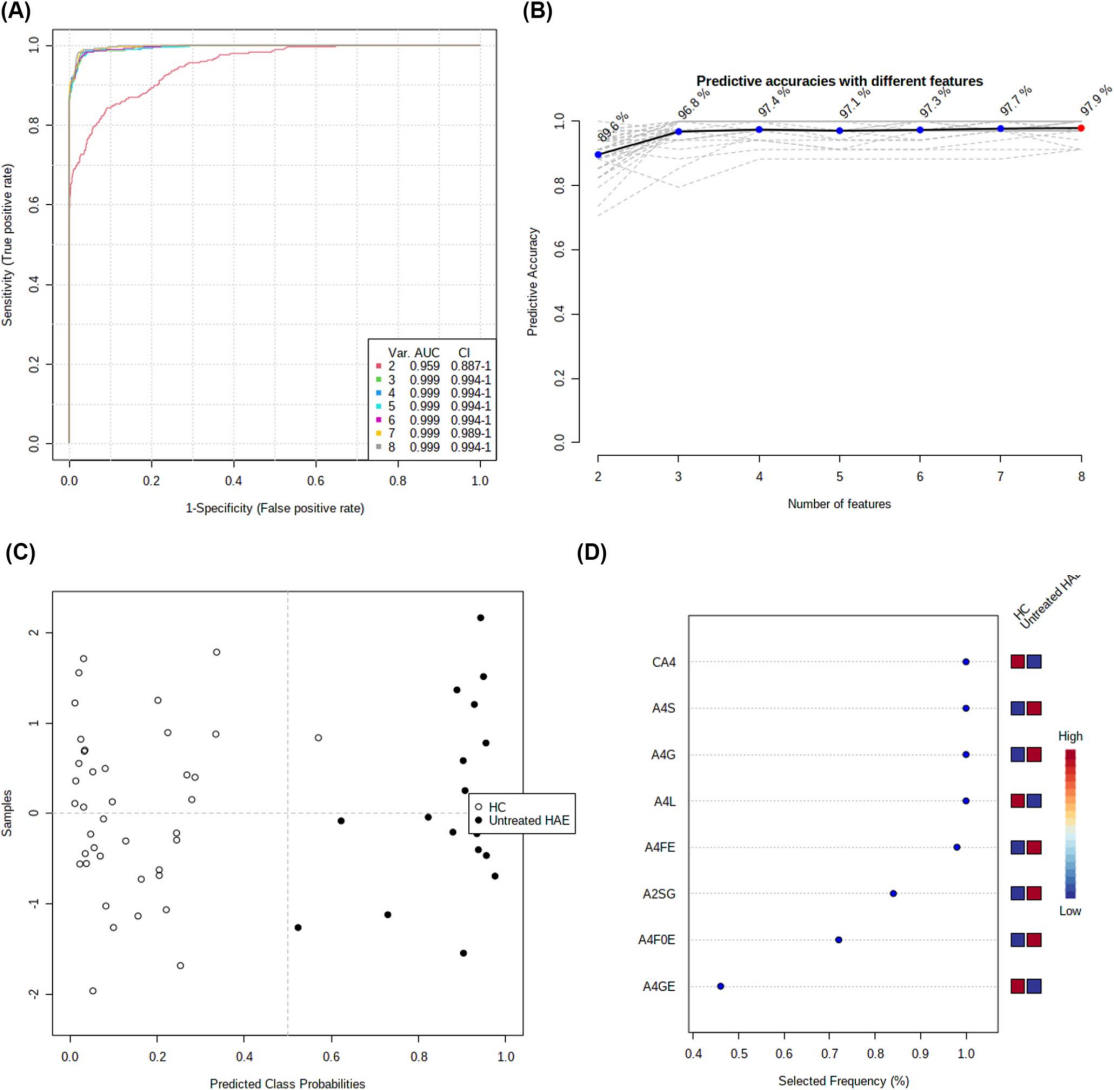
Classification models for differentiating between hereditary angioedema (HAE) and healthy controls based on the eight differentially expressed glycan traits in the validation cohort. (A) Receiver operator characteristics (ROC) curves for the top seven putative biomarker models, based on each model's average performance across all Monte Carlo cross validation (MCCV) runs. (B) Plot of the classification accuracy of the classification models with an increased number of glycan traits. The most accurate model is marked with a red dot. (C) Plot of the classification between case and control using a single biomarker model. Due to the equilibrium of the subsampling, the classification boundary is at the center (*x* = 0.5). Selected model: seven. (D) Plot of the most important glycan traits according to the classification models (from most to least important). HAE, hereditary angioedema; HC, healthy control

The top seven potential classification biomarker models were constructed using multivariate algorithms for SVMs, based on the eight replicated differentially expressed derived *N*‐glycan traits in the two independent cohorts (Figure 3; Figure S2). For each model, two to eight derived *N*‐glycan traits were selected via automated important feature identification (Figure 3D; Figure S2D). ROC curves were generated for each model. The performance of the seven models was highly accurate, with AUCs ranging from 0.927 to 0.931, and predictive accuracies from 87.5% to 90.0%, for discriminating between HAE patients and HCs in the discovery cohort (Figure S2A–S2C). The performance of the models was validated in the validation cohort, with AUCs ranging from 0.959 to 0.999, and predictive accuracies from 89.6% to 97.9% (Figure 3A–3C). Of note, the diagnostic performance of these biomarker models was better than that of individual derived *N*‐glycan traits (Figures S3 and S4).

### 3.3 Plasma *N*‐glycomic changes in HAE patients after treatment

Among the eight replicated abnormal plasma *N*‐glycomic features identified in HAE patients, A4G, A4S, A4L, A4F0E, A4FE, and A4GE showed responses to treatment (Figure 2; Figure S1). After treatment, these derived *N*‐glycan traits showed significant differences between treated and untreated HAE patient groups and returned to near normal levels (Figure 2; Figure S1). In contrast, derived *N*‐glycan traits CA4 and A2SG did not change after treatment (Figure 2; Figure S1). In addition, medication control can reduce the frequency of edema attacks of the patients (data not shown). To investigate whether these glycan traits could be used as biomarkers for disease monitoring/prognosis, we attempted to construct predictive models using multivariate algorithms based on the derived glycans traits that showed a response to treatment. The top five models included two to six of the glycan traits with “accurate” performance based on their AUCs, which ranged from 0.809 to 0.868 in predicting the response to treatment in the discovery cohort (Figure S5A). The results were validated in the validation cohort, with AUCs ranging from 0.831 to 0.852 (Figure S5B). These models may have applications as biomarkers in HAE disease monitoring and prognosis.

### 3.4 Association between plasma *N*‐glycomes and clinical features and parameters

The association between plasma *N*‐glycomes and the occurrence of laryngeal angioedema, gastrointestinal angioedema, and disease severity score in HAE patients was explored by logistic regression. A severity scoring system mainly based on the edema frequency and locations was used to assess the disease severity of HAE patients, as previously described.^20^ Specifically, the clinical severity score (0–7 points) system was performed as follow: frequency of attacks >1/month, 1 point: skin edema of any area ever, 1 point: single submucous site involvement, 1 point: multiple submucous sites involvement, 2 points; emergency visit ever, 1 point; improper abdominal operation ever, 1 point; tracheotomy, 1 point.^20^ Galactosylation of diantennary glycans (A2G) was positively associated with the occurrence of laryngeal angioedema in HAE patients (Table S5). α2,3‐linked sialylation (A2F0L, A2F0GL, and A3L) was negatively associated with gastrointestinal angioedema occurrence (Table S5). Galactosylation of diantennary glycans (A2G) was found to be positively associated with disease severity score (Table S5). Several glycan traits (A3F, A3LF, A3EF, A3F0S, and A4GE) were associated with plasma levels of CI‐INH (Table S5). The associations of these glycan traits were validated in the validation cohort (Table S5).

### 3.5 Performance of plasma glycan traits in predicting the occurrence of laryngeal and gastrointestinal angioedema

**FIGURE 4:**
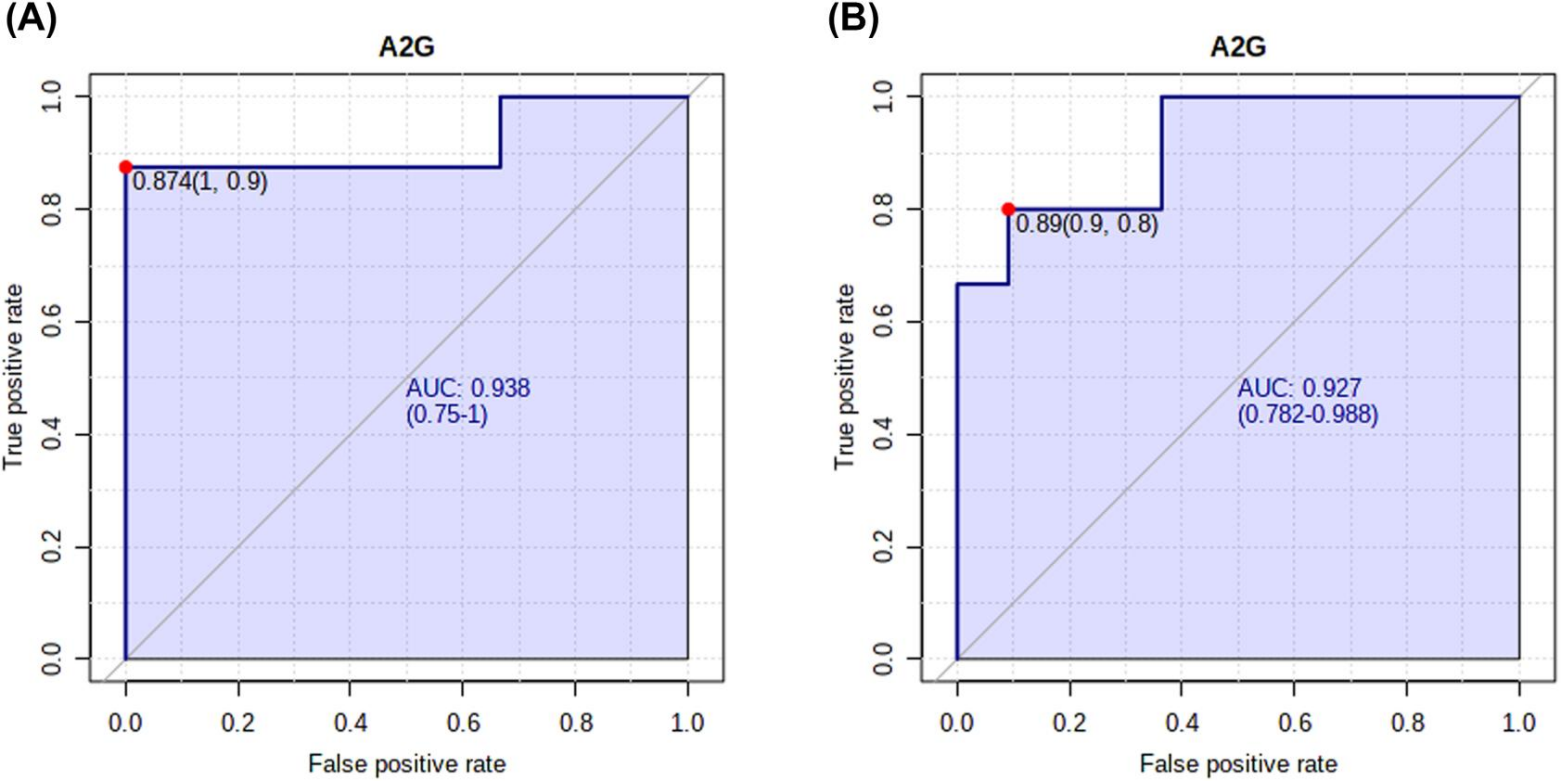
Performance of glycan trait A2G in predicting the occurrence of laryngeal angioedema (A) in the discovery cohort and (B) in the validation cohort. The red dot represents the optimal cut‐off value

The predictive efficacy of glycan traits with strong associations with laryngeal or gastrointestinal angioedema was evaluated by plotting ROC curves. In the discovery cohort, the AUC of A2G was 0.938 for distinguishing between HAE groups in which laryngeal angioedema had ever occurred versus not occurred (Figure 4A). The performance of A2G in predicting the occurrence of laryngeal angioedema was validated in the validation cohort with an AUC of 0.927 (Figure 4B), suggesting that A2G has good predictive ability for laryngeal angioedema occurrence. Based on the three derived glycan traits that showed strong associations with gastrointestinal angioedema, two potential predictive biomarker models were constructed (Figure S6). For each model, two to three derived *N*‐glycan traits were selected through automated important feature identification (Figure S6). ROC curves were generated for each model. The models were moderately accurate (0.7 < AUC < 0.8) in predicting whether HAE patients will ever develop gastrointestinal angioedema (Figure S6).

## 4 DISCUSSION

Accurate, non‐invasive biomarkers for the diagnosis, prediction of disease severity, and disease monitoring of HAE are currently unmet clinical needs. Recently, plasma/serum protein *N*‐glycomes have been identified as biologically significant, and have emerged as a showcase of non‐invasive biomarkers for various diseases.^10^, ^11^, ^12^, ^13^, ^14^, ^18^ The objective of this study was to reveal the disease‐specific *N*‐glycome phenotype of HAE to discover potential biomarkers for the diagnosis, prediction of disease severity, and monitoring of HAE progression. Primary dysregulation of plasma *N*‐glycosylation was identified in HAE patients, particularly in the antennarity of glycans, galactosylation, and (α2,3‐ and α2,6‐linked) sialylation. Eight derived glycan traits were abnormal in HAE patients, from which seven classification models with potential as biomarkers were constructed. Several of these dysregulated glycan traits in HAE returned to near normal levels after treatment, showing potential in monitoring this disease. Predictive models based on the glycan traits that were strongly associated with clinical symptoms had good predictive ability for disease severity. To our knowledge, this study is the first report of the plasma protein *N*‐glycome of HAE, and provides putative glycan‐based biomarkers with validation in an independent cohort. In previous studies of plasma/serum *N*‐glycomes for diseases other than HAE, the differentially expressed *N*‐glycan traits were different from those identified for HAE in the present study,^10^, ^11^, ^12^, ^13^, ^14^ confirming a disease‐specific *N*‐glycome signature, and suggesting that the plasma *N*‐glycome can serve as disease‐specific biomarkers.

Here, we found that the levels of galactosylation of tetra‐antennary glycans (A4G) and galactosylation of sialylated diantennary glycans (A2SG) were increased in HAE compared to that in HCs. Higher galactosylation level was previously linked with increased risk of developing type 2 diabetes.^21^ Moreover, the levels of tri‐ and tetra‐galactosylated *N*‐glycans were previously reported to be increased with age in patients with Down syndrome, but not in HCs.^10^ In humans, the glycan traits A2SG/A4G are primarily derived from alpha1‐antitrypsin, alpha1B‐glycoprotein, fibrinogen, haptoglobin, hemopexin, serotransferrin, and other acute‐phase proteins produced in the liver.^18^ Evidence for altered hepatic synthesis has been found in HAE patients.^22^ Furthermore, some of these liver‐derived proteins are related to the pathophysiology of HAE. Acute‐phase proteins, such as fibrinogen, are risk factors for angioedema induced by bradykinin.^23^ In addition, the production of the inflammatory peptide bradykinin by the contact system can be inhibited by alpha1‐antitrypsin variants.^24^ Galactosylation occurs via a set of beta‐1,4‐galactosyltransferases, the activity of which in plasma is associated with inflammatory diseases and aging.^10^, ^25^ Decreased IgG‐derived galactosylation has been linked to inflammation, immune disorders, cancers, and aging.^26^, ^27^, ^28^ We also observed decreased IgG‐galactosylation in HAE patient plasma, although this decrease was not significant after multiple testing correction (TA2FS0). Though the role of increased non‐IgG‐derived galactosylation, which was linked to acute‐phase liver proteins in the present study, has not yet been established, we assume that abnormal non‐IgG‐derived galactosylation is involved in the causal mechanisms of HAE, and thus warrants further investigation.

Sialylation directly participates in the activation of the immune system, which depends on sialic acid linkage types.^29^, ^30^ For example, siglecs on immune cells specifically recognize non‐fucosylated α2,6‐sialic acid epitopes.^29^ Our novel MS‐based approach enables the discrimination between α2,3‐linked and α2,6‐linked sialylation. Overall, we found sialylation of tetra‐antennary glycans (A4S) was higher in HAE patients than in HCs. Interestingly, sialylation within non‐fucosylated glycans (A4F0E) was increased, whereas sialyation within fucosylated glycans (A4FE) was decreased in HAE patients. The decrease in α2,6‐sialylation with a fucose (A4FE) may derive from immunoglobulins, whereas the increased glycan traits are primarily derived from a mixture of glycoproteins containing highly sialylated glycans produced by the liver (e.g., alpha1‐acid glycoprotein and alpha1‐antitrypsin),^18^ as immunoglobulins primarily carry the α2,6‐sialylation with core‐fucosylation.^31^, ^32^ The observed increase in sialylation within non‐fucosylated liver‐derived tetra‐antennary glycans might reflect glycosylation and abundance changes in acute‐phase proteins. Abnormal sialylation has been found previously in autoimmune diseases, multiple cancer types, and inflammation.^33^, ^34^ Furthermore, α2,6‐sialylation of non‐fucosylated multiple antennary glycans (A(3–4)F0GE) is associated with inflammatory markers in inflammatory bowel disease.^12^ Although most glycan traits containing sialylation were altered during HAE, our data suggest that these changes in sialylation were partially driven by α2,6‐sialyltransferases. ST6Gal1 is the main sialyltransferase, which attaches α2,6‐linked sialic acids to *N*‐glycans.^25^ Circulatory ST6Gal1 is a systemic regulator, and can directly influence the number of inflammatory cells.^35^, ^36^ We hypothesize that abnormal α2,6‐linked sialylation of tetra‐antennary glycans (A4F0E, A4FE, and A4GE) in HAE may be implicated in the HAE‐associated inflammatory changes.

We found a series of glycan traits that showed associations with clinical features or parameters. Considering the stability of glycans during storage, convenience to detect, low amount of plasma required, and low cost, the glycan traits related to clinical parameter (CI‐INH) may serve as biomarkers for HAE diagnosis. Of note, as excellent diagnostic tests (high sensitivity/specificity) for HAE‐C1INH deficiency exist, the value of the potential glycan biomarkers screened out in the present study may be less for diagnosis of HAE‐C1INH deficiency. However, the potential value of the glycan biomarker candidates for the diagnosis of HAE‐normal C1INH (where diagnostics are sorely lacking) would be a strong consideration for future study and work. On the other hand, the glycan traits associated with laryngeal or gastrointestinal angioedema occurrence or disease severity (represented by severity score in the present study) may play a critical role in predicting the disease severity of HAE, and guide disease prevention and treatment. The predictive and prognostic value of these glycan traits for anatomical location of attacks and/or attack frequency would represent an advance since it largely lacks any predictors of phenotype or clinical course in this unpredictable condition in the clinical setting. In the present study, “response to treatment” means glycans returned to (near) normal levels after treatment. Among the eight replicated abnormal plasma *N*‐glycomic features identified in HAE patients, five *N*‐glycan traits showed responses to treatment. After treatment, these derived *N*‐glycan traits showed significant differences between treated and untreated HAE patient groups and returned to near normal levels. Additionally, HAE patients with regular use of danazol >1 month were included in the subgroup of treated HAE. Medication control can reduce the frequency of edema attacks of these patients (data not shown). Considering both the glycan changes and reduction of attacks after treatment, we propose that the *N*‐glycan traits which showed response to treatment may have potential as predictive or monitoring biomarkers. However, this needs further long‐term follow‐up and validation studies in large cohorts.

The present study has some limitations. First, isomers may exist for our assigned glycan structures. Second, MS‐based *N*‐glycome profiling provides relative quantification, and is influenced to some extent by plasma/serum levels of the related glycoproteins. Quantifying protein‐specific glycosylation in combination with measuring the levels of glycoproteins will give in‐depth insights into the mechanisms underpinning the *N*‐glycome, but remains a challenge in this field. Third, because of the rarity of this disease, the untreated and treated HAE groups could not be paired, which could affect the resulting data. Despite these limitations, our research serves as a starting point for future validation and mechanistic studies.

In conclusion, using comprehensive *N*‐glycomic profiling methods, we analyzed the plasma *N*‐glycome for two independent well‐characterized type 1 HAE cohorts, and for the first time reported the full plasma *N*‐glycomic signature of HAE. We identified plasma *N*‐glycosylation changes specific to HAE, and observed major disease‐specific dysregulated glycosylation, namely branching of complex (CA), galactosylation, and sialylation. Novel associations between clinical symptoms/parameters of HAE and glycosylation were found. The combination of altered glycan traits was used to generate classification/prediction models that could be used for the diagnosis, prediction of disease severity, and monitoring of HAE. All the results were validated in an independent validation cohort. These results may play a critical role in predicting/assessing the disease severity of HAE in the future. Further studies are needed to improve our understanding of the role of glycosylation in the pathology of HAE.

## CONFLICT OF INTEREST

The authors declare no conflict of interest.

## Supporting information

Supplementary Material S1
