# Supplementary material for: Validation of diagnostic and predictive biomarkers for hereditary angioedema via plasma N‐glycomics
Source: Clin Transl Allergy. 2021 Dec 27;11(10):e12090. doi: 10.1002/clt2.12090 (PMC8712629; doi:10.1002/clt2.12090)

**Supporting Information**

**Validation of diagnostic and predictive biomarkers for** **hereditary angioedema via plasma *N*-glycomics**

Zejian Zhang^a^, Xue Wang^b^, Jianqing Gu^b^, Jianqiang Wu^a^, Yang Cao^b^, Yingyang Xu^b^, Lisha Li^b^, Kai Guan^b^, Peng Liu^a^, Jia Yin^b^, Yuxiang Zhi^b^*and Shuyang Zhang^c^*

^a^Department of Medical Research Center, State Key Laboratory of Complex Severe and Rare Diseases, Peking Union Medical College Hospital, Chinese Academy of Medical Sciences and Peking Union Medical College, No. 1 Shuaifuyuan Wangfujing, Dongcheng District, Beijing 100730, China

^b^Department of Allergy & Clinical Immunology, National Clinical Research Center for Immunologic Diseases, Peking Union Medical College Hospital, Chinese Academy of Medical Sciences and Peking Union Medical College, No. 1 Shuaifuyuan Wangfujing, Dongcheng District, Beijing, 100730, China

^c^Department of Cardiology, State Key Laboratory of Complex Severe and Rare Diseases, Peking Union Medical College Hospital, Chinese Academy of Medical Sciences and Peking Union Medical College, No. 1 Shuaifuyuan Wangfujing, Dongcheng District, Beijing, 100730, China

*Correspondence should be addressed to:

Yuxiang Zhi, Department of Allergy & Clinical Immunology, National Clinical Research Center for Immunologic Diseases, Peking Union Medical College Hospital, Chinese Academy of Medical Sciences and Peking Union Medical College, No. 1 Shuaifuyuan Wangfujing, Dongcheng District, Beijing, 100730, China. E-mail: yuxiang_zhi@126.com Tel: +86-10-69151601

Shuyang Zhang, Department of Cardiology, State Key Laboratory of Complex Severe and Rare Diseases, Peking Union Medical College Hospital, Chinese Academy of Medical Sciences and Peking Union Medical College, No. 1 Shuaifuyuan Wangfujing, Dongcheng District, Beijing, 100730, China. E-mail: shuyangzhang103@nrdrs.org, Tel: +86-10-69155810

**Supplemental tables**

**Supplemental Table S1. Matrix-assisted laser desorption/ionization time-of-flight mass spectrometry (MALDI-TOF-MS) re-calibrant list (for MassyTools).** *N*-Glycan compositions and theoretical masses [M+Na]^+^ used for internal re-calibration.

| **Composition** | ***m/z* ([M+Na]+, calculated)** |
| --- | --- |
| H3N4F1 | 1485.5337 |
| H4N4F1 | 1647.5865 |
| H5N4F1 | 1809.6393 |
| H5N4E1 | 1982.7081 |
| H5N4E1F1 | 2128.7660 |
| H6N5L1E2 | 2941.0552 |
| H6N5L1E2F1 | 3087.1131 |
| H7N6E1L3 | 3533.2303 |

**Supplemental Table S2. Overview of detected *N*-glycans. Compositions were detected using MALDI-TOF-MS.** The *N*-glycans were released and the *N*-acetylneuraminic acids were derivatized by ethyl esterification. All species are assigned [M+Na]^+^. H = hexose; N = N-acetylhexosamine; F = deoxyhexose (fucose); L = lactonized N-acetylneuraminic acid (α2,3-linked); E = ethyl esterified N-acetylneuraminic acid (α2,6-linked).

| **Observed *m/z*** | **Input composition** | | | | | **Modifiers** | | | **Calculated** | | **Error** | |
| --- | --- | --- | --- | --- | --- | --- | --- | --- | --- | --- | --- | --- |
|  |  |  |  |  |  |  |  |  |  |  |  |  |
|  | H | N | F | L | E | H_2_O | Na+ | Ac | Composition | Mass |  | |
| *m/z* | 162.0528234 | 203.0793725 | 146.0579088 | 273.0848518 | 319.1267166 | 18.01056468 | 22.9892207 | 42.01056 |  | *m/z* | error (Da) | error (ppm) |
|  |  |  |  |  |  |  |  |  |  |  |  |  |
| 1257.42140 | 5 | 2 | 0 | 0 | 0 | 1 | 1 | 0 | H5N2 | 1257.42265 | -0.001 | -1.00 |
| 1419.47650 | 6 | 2 | 0 | 0 | 0 | 1 | 1 | 0 | H6N2 | 1419.47547 | 0.001 | 0.73 |
| 1444.50331 | 4 | 3 | 1 | 0 | 0 | 1 | 1 | 0 | H4N3F1 | 1444.50711 | -0.004 | -2.63 |
| 1455.52382 | 3 | 3 | 0 | 0 | 1 | 1 | 1 | 0 | H3N3E1 | 1455.52309 | 0.001 | 0.50 |
| 1460.50960 | 5 | 3 | 0 | 0 | 0 | 1 | 1 | 0 | H5N3 | 1460.50202 | 0.008 | 5.19 |
| 1485.52557 | 3 | 4 | 1 | 0 | 0 | 1 | 1 | 0 | H3N4F1 | 1485.53365 | -0.008 | -5.44 |
| 1501.53395 | 4 | 4 | 0 | 0 | 0 | 1 | 1 | 0 | H4N4 | 1501.52857 | 0.005 | 3.58 |
| 1542.56454 | 3 | 5 | 0 | 0 | 0 | 1 | 1 | 0 | H3N5 | 1542.55512 | 0.009 | 6.11 |
| 1601.58595 | 3 | 3 | 1 | 0 | 1 | 1 | 1 | 0 | H3N3F1E1 | 1601.58100 | 0.005 | 3.09 |
| 1617.57161 | 4 | 3 | 0 | 0 | 1 | 1 | 1 | 0 | H4N3E1 | 1617.57591 | -0.004 | -2.66 |
| 1622.55751 | 6 | 3 | 0 | 0 | 0 | 1 | 1 | 0 | H6N3 | 1622.55484 | 0.003 | 1.64 |
| 1647.58107 | 4 | 4 | 1 | 0 | 0 | 1 | 1 | 0 | H4N4F1 | 1647.58648 | -0.005 | -3.29 |
| 1663.58083 | 5 | 4 | 0 | 0 | 0 | 1 | 1 | 0 | H5N4 | 1663.58139 | -0.001 | -0.34 |
| 1688.62253 | 3 | 5 | 1 | 0 | 0 | 1 | 1 | 0 | H3N5F1 | 1688.61303 | 0.010 | 5.63 |
| 1704.60239 | 4 | 5 | 0 | 0 | 0 | 1 | 1 | 0 | H4N5 | 1704.60794 | -0.006 | -3.26 |
| 1743.58021 | 8 | 2 | 0 | 0 | 0 | 1 | 1 | 0 | H8N2 | 1743.58112 | -0.001 | -0.52 |
| 1763.62720 | 4 | 3 | 1 | 0 | 1 | 1 | 1 | 0 | H4N3F1E1 | 1763.63382 | -0.007 | -3.76 |
| 1779.63523 | 5 | 3 | 0 | 0 | 1 | 1 | 1 | 0 | H5N3E1 | 1779.62874 | 0.006 | 3.65 |
| 1809.64140 | 5 | 4 | 1 | 0 | 0 | 1 | 1 | 0 | H5N4F1 | 1809.63930 | 0.002 | 1.16 |
| 1820.64849 | 4 | 4 | 0 | 0 | 1 | 1 | 1 | 0 | H4N4E1 | 1820.65529 | -0.007 | -3.73 |
| 1850.66783 | 4 | 5 | 1 | 0 | 0 | 1 | 1 | 0 | H4N5F1 | 1850.66585 | 0.002 | 1.07 |
| 1866.65107 | 5 | 5 | 0 | 0 | 0 | 1 | 1 | 0 | H5N5 | 1866.66077 | -0.010 | -5.19 |
| 1905.63381 | 9 | 2 | 0 | 0 | 0 | 1 | 1 | 0 | H9N2 | 1905.63394 | 0.000 | -0.07 |
| 1936.66355 | 5 | 4 | 0 | 1 | 0 | 1 | 1 | 0 | H5N4L1 | 1936.66624 | -0.003 | -1.39 |
| 1941.67568 | 6 | 3 | 0 | 0 | 1 | 1 | 1 | 0 | H6N3E1 | 1941.68156 | -0.006 | -3.03 |
| 1966.70941 | 4 | 4 | 1 | 0 | 1 | 1 | 1 | 0 | H4N4F1E1 | 1966.71319 | -0.004 | -1.93 |
| 1982.71691 | 5 | 4 | 0 | 0 | 1 | 1 | 1 | 0 | H5N4E1 | 1982.70811 | 0.009 | 4.44 |
| 2012.70797 | 5 | 5 | 1 | 0 | 0 | 1 | 1 | 0 | H5N5F1 | 2012.71867 | -0.011 | -5.32 |
| 2023.73539 | 4 | 5 | 0 | 0 | 1 | 1 | 1 | 0 | H4N5E1 | 2023.73466 | 0.001 | 0.36 |
| 2028.70858 | 6 | 5 | 0 | 0 | 0 | 1 | 1 | 0 | H6N5 | 2028.71359 | -0.005 | -2.47 |
| 2082.71994 | 5 | 4 | 1 | 1 | 0 | 1 | 1 | 0 | H5N4F1L1 | 2082.72415 | -0.004 | -2.02 |
| 2128.76828 | 5 | 4 | 1 | 0 | 1 | 1 | 1 | 0 | H5N4F1E1 | 2128.76602 | 0.002 | 1.06 |
| 2169.79263 | 4 | 5 | 1 | 0 | 1 | 1 | 1 | 0 | H4N5F1E1 | 2169.79257 | 0.000 | 0.03 |
| 2185.77761 | 5 | 5 | 0 | 0 | 1 | 1 | 1 | 0 | H5N5E1 | 2185.78748 | -0.010 | -4.51 |
| 2209.74115 | 5 | 4 | 0 | 2 | 0 | 1 | 1 | 0 | H5N4L2 | 2209.75110 | -0.010 | -4.50 |
| 2255.80135 | 5 | 4 | 0 | 1 | 1 | 1 | 1 | 0 | H5N4L1E1 | 2255.79296 | 0.008 | 3.72 |
| 2274.82676 | 5 | 4 | 2 | 0 | 1 | 1 | 1 | 0 | H5N4F2E1 | 2274.82393 | 0.003 | 1.25 |
| 2301.83186 | 5 | 4 | 0 | 0 | 2 | 1 | 1 | 0 | H5N4E2 | 2301.83483 | -0.003 | -1.29 |
| 2331.85031 | 5 | 5 | 1 | 0 | 1 | 1 | 1 | 0 | H5N5F1E1 | 2331.84539 | 0.005 | 2.11 |
| 2347.84067 | 6 | 5 | 0 | 0 | 1 | 1 | 1 | 0 | H6N5E1 | 2347.84031 | 0.000 | 0.16 |
| 2355.80936 | 5 | 4 | 1 | 2 | 0 | 1 | 1 | 0 | H5N4F1L2 | 2355.80900 | 0.000 | 0.15 |
| 2372.86387 | 4 | 6 | 1 | 0 | 1 | 1 | 1 | 0 | H4N6F1E1 | 2372.87194 | -0.008 | -3.40 |
| 2401.85854 | 5 | 4 | 1 | 1 | 1 | 1 | 1 | 0 | H5N4F1L1E1 | 2401.85087 | 0.008 | 3.19 |
| 2447.90303 | 5 | 4 | 1 | 0 | 2 | 1 | 1 | 0 | H5N4F1E2 | 2447.89273 | 0.010 | 4.21 |
| 2493.90582 | 6 | 5 | 1 | 0 | 1 | 1 | 1 | 0 | H6N5F1E1 | 2493.89821 | 0.008 | 3.05 |
| 2504.90801 | 5 | 5 | 0 | 0 | 2 | 1 | 1 | 0 | H5N5E2 | 2504.91420 | -0.006 | -2.47 |
| 2620.91666 | 6 | 5 | 0 | 1 | 1 | 1 | 1 | 0 | H6N5L1E1 | 2620.92516 | -0.009 | -3.24 |
| 2650.96751 | 5 | 5 | 1 | 0 | 2 | 1 | 1 | 0 | H5N5F1E2 | 2650.97211 | -0.005 | -1.73 |
| 2666.97462 | 6 | 5 | 0 | 0 | 2 | 1 | 1 | 0 | H6N5E2 | 2666.96702 | 0.008 | 2.85 |
| 2766.99163 | 6 | 5 | 1 | 1 | 1 | 1 | 1 | 0 | H6N5F1L1E1 | 2766.98307 | 0.009 | 3.09 |
| 2813.01256 | 6 | 5 | 1 | 0 | 2 | 1 | 1 | 0 | H6N5F1E2 | 2813.02493 | -0.012 | -4.40 |
| 2894.00452 | 6 | 5 | 0 | 2 | 1 | 1 | 1 | 0 | H6N5L2E1 | 2894.01001 | -0.005 | -1.90 |
| 2940.05031 | 6 | 5 | 0 | 1 | 2 | 1 | 1 | 0 | H6N5L1E2 | 2940.05187 | -0.002 | -0.53 |
| 2986.09141 | 6 | 5 | 0 | 0 | 3 | 1 | 1 | 0 | H6N5E3 | 2986.09374 | -0.002 | -0.78 |
| 3040.06430 | 6 | 5 | 1 | 2 | 1 | 1 | 1 | 0 | H6N5F1L2E1 | 3040.06792 | -0.004 | -1.19 |
| 3086.10508 | 6 | 5 | 1 | 1 | 2 | 1 | 1 | 0 | H6N5F1L1E2 | 3086.10978 | -0.005 | -1.52 |
| 3132.14216 | 6 | 5 | 1 | 0 | 3 | 1 | 1 | 0 | H6N5F1E3 | 3132.15165 | -0.009 | -3.03 |
| 3259.14181 | 7 | 6 | 0 | 2 | 1 | 1 | 1 | 0 | H7N6L2E1 | 3259.14220 | 0.000 | -0.12 |
| 3578.27117 | 7 | 6 | 0 | 2 | 2 | 1 | 1 | 0 | H7N6L2E2 | 3578.26892 | 0.002 | 0.63 |

**Supplemental Table S3. Description and calculation of derived glycosylation traits.** M = mannose; Hy = hybrid species; T = within total spectrum; C = within complex species; F = deoxyhexose (fucose); G = galactose; S = N-acetylneuraminic acid (sialic acid); E = α2,6-linked sialic acid; L = α2,3-linked sialic acid; H = hexose (mannose or galactose); N = N-acetylhexosamine (N-acetylglucosamine: GlcNAc).

| **Derived trait** | **Description** | **Formula of calculation** |
| --- | --- | --- |
| **Glycan type** |  |  |
| TM | Relative abundance of high mannose type glycans within total spectrum | TM = ( H5N2 + H6N2 + H8N2 + H9N2 ) / ( H5N2 + H6N2 + H4N3F1 + H3N3E1 + H5N3 + H3N4F1 + H4N4 + H3N5 + H3N3F1E1 + H4N3E1 + H6N3 + H4N4F1 + H5N4 + H3N5F1 + H4N5 + H8N2 + H4N3F1E1 + H5N3E1 + H5N4F1 + H4N4E1 + H4N5F1 + H5N5 + H9N2 + H5N4L1 + H6N3E1 + H4N4F1E1 + H5N4E1 + H5N5F1 + H4N5E1 + H6N5 + H5N4F1L1 + H5N4F1E1 + H4N5F1E1 + H5N5E1 + H5N4L2 + H5N4E1L1 + H5N4F2E1 + H5N4E2 + H5N5F1E1 + H6N5E1 + H5N4F1L2 + H4N6F1E1 + H5N4F1E1L1 + H5N4F1E2 + H6N5F1E1 + H5N5E2 + H6N5E1L1 + H5N5F1E2 + H6N5E2 + H6N5F1E1L1 + H6N5F1E2 + H6N5E1L2 + H6N5E2L1 + H6N5E3 + H6N5F1E1L2 + H6N5F1E2L1 + H6N5F1E3 + H7N6E1L2 + H7N6E2L2) |
| THy | Relative abundance of hybrid type glycans within total spectrum | THy = ( H5N3 + H6N3 + H5N3E1 + H6N3E1 ) / ( H5N2 + H6N2 + H4N3F1 + H3N3E1 + H5N3 + H3N4F1 + H4N4 + H3N5 + H3N3F1E1 + H4N3E1 + H6N3 + H4N4F1 + H5N4 + H3N5F1 + H4N5 + H8N2 + H4N3F1E1 + H5N3E1 + H5N4F1 + H4N4E1 + H4N5F1 + H5N5 + H9N2 + H5N4L1 + H6N3E1 + H4N4F1E1 + H5N4E1 + H5N5F1 + H4N5E1 + H6N5 + H5N4F1L1 + H5N4F1E1 + H4N5F1E1 + H5N5E1 + H5N4L2 + H5N4E1L1 + H5N4F2E1 + H5N4E2 + H5N5F1E1 + H6N5E1 + H5N4F1L2 + H4N6F1E1 + H5N4F1E1L1 + H5N4F1E2 + H6N5F1E1 + H5N5E2 + H6N5E1L1 + H5N5F1E2 + H6N5E2 + H6N5F1E1L1 + H6N5F1E2 + H6N5E1L2 + H6N5E2L1 + H6N5E3 + H6N5F1E1L2 + H6N5F1E2L1 + H6N5F1E3 + H7N6E1L2 + H7N6E2L2 ) |
| MHy | The ratio of high-mannose to hybrid glycans | MHy = (H5N2 + H6N2 + H8N2 + H9N2) / (H5N3 + H6N3 + H5N3E1 + H6N3E1) |
| MM | Average number of mannoses on high mannose type glycans | MM = (5 * (H5N2) + 6 * (H6N2) + 7 * (0) + 8 * ( H8N2 ) + 9 * ( H9N2 ) + 10 * (0) ) / ( H5N2 + H6N2 + H8N2 + H9N2 ) |
| CA1 | Relative abundance of monoantennary glycans within complex type glycans | CA1 = ( H3N3E1 + H3N3F1E1 + H4N3E1 + H4N3F1E1 ) / ( H3N3E1 + H3N4F1 + H4N4 + H3N5 + H3N3F1E1 + H4N3E1 + H4N4F1 + H5N4 + H3N5F1 + H4N5 + H4N3F1E1 + H5N4F1 + H4N4E1 + H4N5F1 + H5N5 + H5N4L1 + H4N4F1E1 + H5N4E1 + H5N5F1 + H4N5E1 + H6N5 + H5N4F1L1 + H5N4F1E1 + H4N5F1E1 + H5N5E1 + H5N4L2 + H5N4E1L1 + H5N4F2E1 + H5N4E2 + H5N5F1E1 + H6N5E1 + H5N4F1L2 + H4N6F1E1 + H5N4F1E1L1 + H5N4F1E2 + H6N5F1E1 + H5N5E2 + H6N5E1L1 + H5N5F1E2 + H6N5E2 + H6N5F1E1L1 + H6N5F1E2 + H6N5E1L2 + H6N5E2L1 + H6N5E3 + H6N5F1E1L2 + H6N5F1E2L1 + H6N5F1E3 + H7N6E1L2 + H7N6E2L2 ) |
| CA2 | Relative abundance of diantennary glycans within complex type glycans | CA2 = ( H3N4F1 + H4N4 + H3N5 + H4N4F1 + H5N4 + H3N5F1 + H4N5 + H5N4F1 + H4N4E1 + H4N5F1 + H5N5 + H5N4L1 + H4N4F1E1 + H5N4E1 + H5N5F1 + H4N5E1 + H5N4F1L1 + H5N4F1E1 + H4N5F1E1 + H5N5E1 + H5N4L2 + H5N4E1L1 + H5N4F2E1 + H5N4E2 + H5N5F1E1 + H5N4F1L2 + H5N4F1E1L1 + H5N4F1E2 + H5N5E2 + H5N5F1E2 ) / ( H3N3E1 + H3N4F1 + H4N4 + H3N5 + H3N3F1E1 + H4N3E1 + H4N4F1 + H5N4 + H3N5F1 + H4N5 + H4N3F1E1 + H5N4F1 + H4N4E1 + H4N5F1 + H5N5 + H5N4L1 + H4N4F1E1 + H5N4E1 + H5N5F1 + H4N5E1 + H6N5 + H5N4F1L1 + H5N4F1E1 + H4N5F1E1 + H5N5E1 + H5N4L2 + H5N4E1L1 + H5N4F2E1 + H5N4E2 + H5N5F1E1 + H6N5E1 + H5N4F1L2 + H4N6F1E1 + H5N4F1E1L1 + H5N4F1E2 + H6N5F1E1 + H5N5E2 + H6N5E1L1 + H5N5F1E2 + H6N5E2 + H6N5F1E1L1 + H6N5F1E2 + H6N5E1L2 + H6N5E2L1 + H6N5E3 + H6N5F1E1L2 + H6N5F1E2L1 + H6N5F1E3 + H7N6E1L2 + H7N6E2L2 ) |
| CA3 | Relative abundance of triantennary glycans within complex type glycans | CA3 = ( H6N5 + H6N5E1 + H6N5F1E1 + H6N5E1L1 + H6N5E2 + H6N5F1E1L1 + H6N5F1E2 + H6N5E1L2 + H6N5E2L1 + H6N5E3 + H6N5F1E1L2 + H6N5F1E2L1 + H6N5F1E3 ) / ( H3N3E1 + H3N4F1 + H4N4 + H3N5 + H3N3F1E1 + H4N3E1 + H4N4F1 + H5N4 + H3N5F1 + H4N5 + H4N3F1E1 + H5N4F1 + H4N4E1 + H4N5F1 + H5N5 + H5N4L1 + H4N4F1E1 + H5N4E1 + H5N5F1 + H4N5E1 + H6N5 + H5N4F1L1 + H5N4F1E1 + H4N5F1E1 + H5N5E1 + H5N4L2 + H5N4E1L1 + H5N4F2E1 + H5N4E2 + H5N5F1E1 + H6N5E1 + H5N4F1L2 + H4N6F1E1 + H5N4F1E1L1 + H5N4F1E2 + H6N5F1E1 + H5N5E2 + H6N5E1L1 + H5N5F1E2 + H6N5E2 + H6N5F1E1L1 + H6N5F1E2 + H6N5E1L2 + H6N5E2L1 + H6N5E3 + H6N5F1E1L2 + H6N5F1E2L1 + H6N5F1E3 + H7N6E1L2 + H7N6E2L2 ) |
| CA4 | Relative abundance of tetra-antennary glycans within complex type glycans | CA4 = ( H4N6F1E1 + H7N6E1L2 + H7N6E2L2 ) / ( H3N3E1 + H3N4F1 + H4N4 + H3N5 + H3N3F1E1 + H4N3E1 + H4N4F1 + H5N4 + H3N5F1 + H4N5 + H4N3F1E1 + H5N4F1 + H4N4E1 + H4N5F1 + H5N5 + H5N4L1 + H4N4F1E1 + H5N4E1 + H5N5F1 + H4N5E1 + H6N5 + H5N4F1L1 + H5N4F1E1 + H4N5F1E1 + H5N5E1 + H5N4L2 + H5N4E1L1 + H5N4F2E1 + H5N4E2 + H5N5F1E1 + H6N5E1 + H5N4F1L2 + H4N6F1E1 + H5N4F1E1L1 + H5N4F1E2 + H6N5F1E1 + H5N5E2 + H6N5E1L1 + H5N5F1E2 + H6N5E2 + H6N5F1E1L1 + H6N5F1E2 + H6N5E1L2 + H6N5E2L1 + H6N5E3 + H6N5F1E1L2 + H6N5F1E2L1 + H6N5F1E3 + H7N6E1L2 + H7N6E2L2 ) |
| **Fucosylation (F)** |  |  |
| CF | Fucosylation within complex type glycans | CF = ( H3N4F1 + H3N3F1E1 + H4N4F1 + H3N5F1 + H4N3F1E1 + H5N4F1 + H4N5F1 + H4N4F1E1 + H5N5F1 + H5N4F1L1 + H5N4F1E1 + H4N5F1E1 + H5N4F2E1 + H5N5F1E1 + H5N4F1L2 + H4N6F1E1 + H5N4F1E1L1 + H5N4F1E2 + H6N5F1E1 + H5N5F1E2 + H6N5F1E1L1 + H6N5F1E2 + H6N5F1E1L2 + H6N5F1E2L1 + H6N5F1E3 ) / ( H3N3E1 + H3N4F1 + H4N4 + H3N5 + H3N3F1E1 + H4N3E1 + H4N4F1 + H5N4 + H3N5F1 + H4N5 + H4N3F1E1 + H5N4F1 + H4N4E1 + H4N5F1 + H5N5 + H5N4L1 + H4N4F1E1 + H5N4E1 + H5N5F1 + H4N5E1 + H6N5 + H5N4F1L1 + H5N4F1E1 + H4N5F1E1 + H5N5E1 + H5N4L2 + H5N4E1L1 + H5N4F2E1 + H5N4E2 + H5N5F1E1 + H6N5E1 + H5N4F1L2 + H4N6F1E1 + H5N4F1E1L1 + H5N4F1E2 + H6N5F1E1 + H5N5E2 + H6N5E1L1 + H5N5F1E2 + H6N5E2 + H6N5F1E1L1 + H6N5F1E2 + H6N5E1L2 + H6N5E2L1 + H6N5E3 + H6N5F1E1L2 + H6N5F1E2L1 + H6N5F1E3 + H7N6E1L2 + H7N6E2L2 ) |
| A1F | Fucosylation within monoantennary glycans | A1F = ( H3N3F1E1 + H4N3F1E1 ) / ( H3N3E1 + H3N3F1E1 + H4N3E1 + H4N3F1E1 ) |
| A2F | Fucosylation within diantennary glycans | A2F = ( H3N4F1 + H4N4F1 + H3N5F1 + H5N4F1 + H4N5F1 + H4N4F1E1 + H5N5F1 + H5N4F1L1 + H5N4F1E1 + H4N5F1E1 + H5N4F2E1 + H5N5F1E1 + H5N4F1L2 + H5N4F1E1L1 + H5N4F1E2 + H5N5F1E2 ) / ( H3N4F1 + H4N4 + H3N5 + H4N4F1 + H5N4 + H3N5F1 + H4N5 + H5N4F1 + H4N4E1 + H4N5F1 + H5N5 + H5N4L1 + H4N4F1E1 + H5N4E1 + H5N5F1 + H4N5E1 + H5N4F1L1 + H5N4F1E1 + H4N5F1E1 + H5N5E1 + H5N4L2 + H5N4E1L1 + H5N4F2E1 + H5N4E2 + H5N5F1E1 + H5N4F1L2 + H5N4F1E1L1 + H5N4F1E2 + H5N5E2 + H5N5F1E2 ) |
| A3F | Fucosylation within triantennary glycans | A3F = ( H6N5F1E1 + H6N5F1E1L1 + H6N5F1E2 + H6N5F1E1L2 + H6N5F1E2L1 + H6N5F1E3 ) / ( H6N5 + H6N5E1 + H6N5F1E1 + H6N5E1L1 + H6N5E2 + H6N5F1E1L1 + H6N5F1E2 + H6N5E1L2 + H6N5E2L1 + H6N5E3 + H6N5F1E1L2 + H6N5F1E2L1 + H6N5F1E3 ) |
| A2S0F | Fucosylation within non-sialylated diantennary glycans | A2S0F = ( H3N4F1 + H4N4F1 + H3N5F1 + H5N4F1 + H4N5F1 + H5N5F1 ) / ( H3N4F1 + H4N4 + H3N5 + H4N4F1 + H5N4 + H3N5F1 + H4N5 + H5N4F1 + H4N5F1 + H5N5 + H5N5F1 ) |
| A2L0F | Fucosylation within diantennary glycans without α2,3-linked sialic acid | A2L0F = ( H3N4F1 + H4N4F1 + H3N5F1 + H5N4F1 + H4N5F1 + H4N4F1E1 + H5N5F1 + H5N4F1E1 + H4N5F1E1 + H5N4F2E1 + H5N5F1E1 + H5N4F1E2 + H5N5F1E2 ) / ( H3N4F1 + H4N4 + H3N5 + H4N4F1 + H5N4 + H3N5F1 + H4N5 + H5N4F1 + H4N4E1 + H4N5F1 + H5N5 + H4N4F1E1 + H5N4E1 + H5N5F1 + H4N5E1 + H5N4F1E1 + H4N5F1E1 + H5N5E1 + H5N4F2E1 + H5N4E2 + H5N5F1E1 + H5N4F1E2 + H5N5E2 + H5N5F1E2 ) |
| A3L0F | Fucosylation within triantennary glycans without α2,3-linked sialic acid | A3L0F = ( H6N5F1E1 + H6N5F1E2 + H6N5F1E3 ) / ( H6N5 + H6N5E1 + H6N5F1E1 + H6N5E2 + H6N5F1E2 + H6N5E3 + H6N5F1E3 ) |
| A2E0F | Fucosylation within diantennary glycans without α2,6-linked sialic acid | A2E0F = ( H3N4F1 + H4N4F1 + H3N5F1 + H5N4F1 + H4N5F1 + H5N5F1 + H5N4F1L1 + H5N4F1L2 ) / ( H3N4F1 + H4N4 + H3N5 + H4N4F1 + H5N4 + H3N5F1 + H4N5 + H5N4F1 + H4N5F1 + H5N5 + H5N4L1 + H5N5F1 + H5N4F1L1 + H5N4L2 + H5N4F1L2 ) |
| A2SF | Fucosylation within sialylated diantennary glycans | A2SF = ( H4N4F1E1 + H5N4F1L1 + H5N4F1E1 + H4N5F1E1 + H5N4F2E1 + H5N5F1E1 + H5N4F1L2 + H5N4F1E1L1 + H5N4F1E2 + H5N5F1E2 ) / ( H4N4E1 + H5N4L1 + H4N4F1E1 + H5N4E1 + H4N5E1 + H5N4F1L1 + H5N4F1E1 + H4N5F1E1 + H5N5E1 + H5N4L2 + H5N4E1L1 + H5N4F2E1 + H5N4E2 + H5N5F1E1 + H5N4F1L2 + H5N4F1E1L1 + H5N4F1E2 + H5N5E2 + H5N5F1E2 ) |
| A2LF | Fucosylation within diantennary glycans with α2,3-linked sialic acid | A2LF = ( H5N4F1L1 + H5N4F1L2 + H5N4F1E1L1 ) / ( H5N4L1 + H5N4F1L1 + H5N4L2 + H5N4E1L1 + H5N4F1L2 + H5N4F1E1L1 ) |
| A3LF | Fucosylation within triantennary glycans with α2,3-linked sialic acid | A3LF = ( H6N5F1E1L1 + H6N5F1E1L2 + H6N5F1E2L1 ) / ( H6N5E1L1 + H6N5F1E1L1 + H6N5E1L2 + H6N5E2L1 + H6N5F1E1L2 + H6N5F1E2L1 ) |
| A2EF | Fucosylation within diantennary glycans with α2,6-linked sialic acid | A2EF = ( H4N4F1E1 + H5N4F1E1 + H4N5F1E1 + H5N4F2E1 + H5N5F1E1 + H5N4F1E1L1 + H5N4F1E2 + H5N5F1E2 ) / ( H4N4E1 + H4N4F1E1 + H5N4E1 + H4N5E1 + H5N4F1E1 + H4N5F1E1 + H5N5E1 + H5N4E1L1 + H5N4F2E1 + H5N4E2 + H5N5F1E1 + H5N4F1E1L1 + H5N4F1E2 + H5N5E2 + H5N5F1E2 ) |
| A3EF | Fucosylation within triantennary glycans with α2,6-linked sialic acid | A3EF = ( H6N5F1E1 + H6N5F1E1L1 + H6N5F1E2 + H6N5F1E1L2 + H6N5F1E2L1 + H6N5F1E3 ) / ( H6N5E1 + H6N5F1E1 + H6N5E1L1 + H6N5E2 + H6N5F1E1L1 + H6N5F1E2 + H6N5E1L2 + H6N5E2L1 + H6N5E3 + H6N5F1E1L2 + H6N5F1E2L1 + H6N5F1E3 ) |
| **Antennary fucosylation (Fa)** |  |  |
| CFa | Relative abundance of species with 2 fucoses (i.e. at least one antennary fucose) within all complex type glycans | CFa = ( H5N4F2E1 ) / ( H3N3E1 + H3N4F1 + H4N4 + H3N5 + H3N3F1E1 + H4N3E1 + H4N4F1 + H5N4 + H3N5F1 + H4N5 + H4N3F1E1 + H5N4F1 + H4N4E1 + H4N5F1 + H5N5 + H5N4L1 + H4N4F1E1 + H5N4E1 + H5N5F1 + H4N5E1 + H6N5 + H5N4F1L1 + H5N4F1E1 + H4N5F1E1 + H5N5E1 + H5N4L2 + H5N4E1L1 + H5N4F2E1 + H5N4E2 + H5N5F1E1 + H6N5E1 + H5N4F1L2 + H4N6F1E1 + H5N4F1E1L1 + H5N4F1E2 + H6N5F1E1 + H5N5E2 + H6N5E1L1 + H5N5F1E2 + H6N5E2 + H6N5F1E1L1 + H6N5F1E2 + H6N5E1L2 + H6N5E2L1 + H6N5E3 + H6N5F1E1L2 + H6N5F1E2L1 + H6N5F1E3 + H7N6E1L2 + H7N6E2L2 ) |
| A2Fa | Relative abundance of species with 2 fucoses (i.e. at least one antennary fucose) within diantennary glycans | A2Fa = ( H5N4F2E1 ) / ( H3N4F1 + H4N4 + H3N5 + H4N4F1 + H5N4 + H3N5F1 + H4N5 + H5N4F1 + H4N4E1 + H4N5F1 + H5N5 + H5N4L1 + H4N4F1E1 + H5N4E1 + H5N5F1 + H4N5E1 + H5N4F1L1 + H5N4F1E1 + H4N5F1E1 + H5N5E1 + H5N4L2 + H5N4E1L1 + H5N4F2E1 + H5N4E2 + H5N5F1E1 + H5N4F1L2 + H5N4F1E1L1 + H5N4F1E2 + H5N5E2 + H5N5F1E2 ) |
| **Bisection (B)** |  |  |
| CB | Relative abundance of species with a bisecting GlcNAc within all complex glycans | CB = ( H3N5 + H3N5F1 + H4N5 + H4N5F1 + H5N5 + H5N5F1 + H4N5E1 + H4N5F1E1 + H5N5E1 + H5N5F1E1 + H5N5E2 + H5N5F1E2 ) / ( H3N3E1 + H3N4F1 + H4N4 + H3N5 + H3N3F1E1 + H4N3E1 + H4N4F1 + H5N4 + H3N5F1 + H4N5 + H4N3F1E1 + H5N4F1 + H4N4E1 + H4N5F1 + H5N5 + H5N4L1 + H4N4F1E1 + H5N4E1 + H5N5F1 + H4N5E1 + H6N5 + H5N4F1L1 + H5N4F1E1 + H4N5F1E1 + H5N5E1 + H5N4L2 + H5N4E1L1 + H5N4F2E1 + H5N4E2 + H5N5F1E1 + H6N5E1 + H5N4F1L2 + H4N6F1E1 + H5N4F1E1L1 + H5N4F1E2 + H6N5F1E1 + H5N5E2 + H6N5E1L1 + H5N5F1E2 + H6N5E2 + H6N5F1E1L1 + H6N5F1E2 + H6N5E1L2 + H6N5E2L1 + H6N5E3 + H6N5F1E1L2 + H6N5F1E2L1 + H6N5F1E3 + H7N6E1L2 + H7N6E2L2 ) |
| A2B | Relative abundance of species with a bisecting GlcNAc within diantennary glycans | A2B = ( H3N5 + H3N5F1 + H4N5 + H4N5F1 + H5N5 + H5N5F1 + H4N5E1 + H4N5F1E1 + H5N5E1 + H5N5F1E1 + H5N5E2 + H5N5F1E2 ) / ( H3N4F1 + H4N4 + H3N5 + H4N4F1 + H5N4 + H3N5F1 + H4N5 + H5N4F1 + H4N4E1 + H4N5F1 + H5N5 + H5N4L1 + H4N4F1E1 + H5N4E1 + H5N5F1 + H4N5E1 + H5N4F1L1 + H5N4F1E1 + H4N5F1E1 + H5N5E1 + H5N4L2 + H5N4E1L1 + H5N4F2E1 + H5N4E2 + H5N5F1E1 + H5N4F1L2 + H5N4F1E1L1 + H5N4F1E2 + H5N5E2 + H5N5F1E2 ) |
| A2F0B | Relative abundance of species with a bisecting GlcNAc within non-fucosylated diantennary glycans | A2F0B = ( H3N5 + H4N5 + H5N5 + H4N5E1 + H5N5E1 + H5N5E2 ) / ( H4N4 + H3N5 + H5N4 + H4N5 + H4N4E1 + H5N5 + H5N4L1 + H5N4E1 + H4N5E1 + H5N5E1 + H5N4L2 + H5N4E1L1 + H5N4E2 + H5N5E2 ) |
| A2FB | Relative abundance of species with a bisecting GlcNAc within fucosylated diantennary | A2FB = ( H3N5F1 + H4N5F1 + H5N5F1 + H4N5F1E1 + H5N5F1E1 + H5N5F1E2 ) / ( H3N4F1 + H4N4F1 + H3N5F1 + H5N4F1 + H4N5F1 + H4N4F1E1 + H5N5F1 + H5N4F1L1 + H5N4F1E1 + H4N5F1E1 + H5N4F2E1 + H5N5F1E1 + H5N4F1L2 + H5N4F1E1L1 + H5N4F1E2 + H5N5F1E2 ) |
| A2S0B | Relative abundance of species with a bisecting GlcNAc within non-sialylated diantennary glycans | A2S0B = ( H3N5 + H3N5F1 + H4N5 + H4N5F1 + H5N5 + H5N5F1 ) / ( H3N4F1 + H4N4 + H3N5 + H4N4F1 + H5N4 + H3N5F1 + H4N5 + H5N4F1 + H4N5F1 + H5N5 + H5N5F1 ) |
| A2SB | Relative abundance of species with a bisecting GlcNAc within sialylated diantennary glycans | A2SB = ( H4N5E1 + H4N5F1E1 + H5N5E1 + H5N5F1E1 + H5N5E2 + H5N5F1E2 ) / ( H4N4E1 + H5N4L1 + H4N4F1E1 + H5N4E1 + H4N5E1 + H5N4F1L1 + H5N4F1E1 + H4N5F1E1 + H5N5E1 + H5N4L2 + H5N4E1L1 + H5N4F2E1 + H5N4E2 + H5N5F1E1 + H5N4F1L2 + H5N4F1E1L1 + H5N4F1E2 + H5N5E2 + H5N5F1E2 ) |
| A2F0S0B | Relative abundance of species with a bisecting GlcNAc within non-fucosylated non-sialylated diantennary glycans | A2F0S0B = ( H3N5 + H4N5 + H5N5 ) / ( H4N4 + H3N5 + H5N4 + H4N5 + H5N5 ) |
| A2F0SB | Relative abundance of species with a bisecting GlcNAc within non-fucosylated sialylated diantennary glycans | A2F0SB = ( H4N5E1 + H5N5E1 + H5N5E2 ) / ( H4N4E1 + H5N4L1 + H5N4E1 + H4N5E1 + H5N5E1 + H5N4L2 + H5N4E1L1 + H5N4E2 + H5N5E2 ) |
| A2FS0B | Relative abundance of species with a bisecting GlcNAc within fucosylated non-sialylated diantennary glycans | A2FS0B = ( H3N5F1 + H4N5F1 + H5N5F1 ) / ( H3N4F1 + H4N4F1 + H3N5F1 + H5N4F1 + H4N5F1 + H5N5F1 ) |
| A2FSB | Relative abundance of species with a bisecting GlcNAc within fucosylated sialylated diantennary glycans | A2FSB = ( H4N5F1E1 + H5N5F1E1 + H5N5F1E2 ) / ( H4N4F1E1 + H5N4F1L1 + H5N4F1E1 + H4N5F1E1 + H5N4F2E1 + H5N5F1E1 + H5N4F1L2 + H5N4F1E1L1 + H5N4F1E2 + H5N5F1E2 ) |
| **Galactosylation (G)** |  |  |
| CG | Galactosylation within all complex glycans | CG = ( H3N3E1 + H4N4 + H3N3F1E1 + H4N3E1 + H4N4F1 + H5N4 + H4N5 + H4N3F1E1 + H5N4F1 + H4N4E1 + H4N5F1 + H5N5 + H5N4L1 + H4N4F1E1 + H5N4E1 + H5N5F1 + H4N5E1 + H6N5 + H5N4F1L1 + H5N4F1E1 + H4N5F1E1 + H5N5E1 + H5N4L2 + H5N4E1L1 + H5N4F2E1 + H5N4E2 + H5N5F1E1 + H6N5E1 + H5N4F1L2 + H4N6F1E1 + H5N4F1E1L1 + H5N4F1E2 + H6N5F1E1 + H5N5E2 + H6N5E1L1 + H5N5F1E2 + H6N5E2 + H6N5F1E1L1 + H6N5F1E2 + H6N5E1L2 + H6N5E2L1 + H6N5E3 + H6N5F1E1L2 + H6N5F1E2L1 + H6N5F1E3 + H7N6E1L2 + H7N6E2L2 ) / ( H3N3E1 + H3N4F1 + H4N4 + H3N5 + H3N3F1E1 + H4N3E1 + H4N4F1 + H5N4 + H3N5F1 + H4N5 + H4N3F1E1 + H5N4F1 + H4N4E1 + H4N5F1 + H5N5 + H5N4L1 + H4N4F1E1 + H5N4E1 + H5N5F1 + H4N5E1 + H6N5 + H5N4F1L1 + H5N4F1E1 + H4N5F1E1 + H5N5E1 + H5N4L2 + H5N4E1L1 + H5N4F2E1 + H5N4E2 + H5N5F1E1 + H6N5E1 + H5N4F1L2 + H4N6F1E1 + H5N4F1E1L1 + H5N4F1E2 + H6N5F1E1 + H5N5E2 + H6N5E1L1 + H5N5F1E2 + H6N5E2 + H6N5F1E1L1 + H6N5F1E2 + H6N5E1L2 + H6N5E2L1 + H6N5E3 + H6N5F1E1L2 + H6N5F1E2L1 + H6N5F1E3 + H7N6E1L2 + H7N6E2L2 ) |
| A2G | Galactosylation per antenna within diantennary glycans | A2G = ( 0/2 * ( H3N4F1 + H3N5 + H3N5F1 ) + 1/2 * ( H4N4 + H4N4F1 + H4N5 + H4N4E1 + H4N5F1 + H4N4F1E1 + H4N5E1 + H4N5F1E1 ) + 2/2 * ( H5N4 + H5N4F1 + H5N5 + H5N4L1 + H5N4E1 + H5N5F1 + H5N4F1L1 + H5N4F1E1 + H5N5E1 + H5N4L2 + H5N4E1L1 + H5N4F2E1 + H5N4E2 + H5N5F1E1 + H5N4F1L2 + H5N4F1E1L1 + H5N4F1E2 + H5N5E2 + H5N5F1E2 ) ) / ( H3N4F1 + H4N4 + H3N5 + H4N4F1 + H5N4 + H3N5F1 + H4N5 + H5N4F1 + H4N4E1 + H4N5F1 + H5N5 + H5N4L1 + H4N4F1E1 + H5N4E1 + H5N5F1 + H4N5E1 + H5N4F1L1 + H5N4F1E1 + H4N5F1E1 + H5N5E1 + H5N4L2 + H5N4E1L1 + H5N4F2E1 + H5N4E2 + H5N5F1E1 + H5N4F1L2 + H5N4F1E1L1 + H5N4F1E2 + H5N5E2 + H5N5F1E2 ) |
| A4G | Galactosylation per antenna within tetra-antennary glycans | A4G = ( 0/4 * (0) + 1/4 * ( H4N6F1E1 ) + 2/4 * (0) + 3/4 * (0) + 4/4 * ( H7N6E1L2 + H7N6E2L2 ) ) / ( H4N6F1E1 + H7N6E1L2 + H7N6E2L2 ) |
| A2F0G | Galactosylation per antenna within non-fucosylated diantennary glycans | A2F0G = ( 0/2 * ( H3N5 ) + 1/2 * ( H4N4 + H4N5 + H4N4E1 + H4N5E1 ) + 2/2 * ( H5N4 + H5N5 + H5N4L1 + H5N4E1 + H5N5E1 + H5N4L2 + H5N4E1L1 + H5N4E2 + H5N5E2 ) ) / ( H4N4 + H3N5 + H5N4 + H4N5 + H4N4E1 + H5N5 + H5N4L1 + H5N4E1 + H4N5E1 + H5N5E1 + H5N4L2 + H5N4E1L1 + H5N4E2 + H5N5E2 ) |
| A2FG | Galactosylation per antenna within fucosylated diantennary glycans | A2FG = ( 0/2 * ( H3N4F1 + H3N5F1 ) + 1/2 * ( H4N4F1 + H4N5F1 + H4N4F1E1 + H4N5F1E1 ) + 2/2 * ( H5N4F1 + H5N5F1 + H5N4F1L1 + H5N4F1E1 + H5N4F2E1 + H5N5F1E1 + H5N4F1L2 + H5N4F1E1L1 + H5N4F1E2 + H5N5F1E2 ) ) / ( H3N4F1 + H4N4F1 + H3N5F1 + H5N4F1 + H4N5F1 + H4N4F1E1 + H5N5F1 + H5N4F1L1 + H5N4F1E1 + H4N5F1E1 + H5N4F2E1 + H5N5F1E1 + H5N4F1L2 + H5N4F1E1L1 + H5N4F1E2 + H5N5F1E2 ) |
| A2S0G | Galactosylation per antenna within non-sialylated diantennary glycans | A2S0G = ( 0/2 * ( H3N4F1 + H3N5 + H3N5F1 ) + 1/2 * ( H4N4 + H4N4F1 + H4N5 + H4N5F1 ) + 2/2 * ( H5N4 + H5N4F1 + H5N5 + H5N5F1 ) ) / ( H3N4F1 + H4N4 + H3N5 + H4N4F1 + H5N4 + H3N5F1 + H4N5 + H5N4F1 + H4N5F1 + H5N5 + H5N5F1 ) |
| A2SG | Galactosylation per antenna within sialylated diantennary glycans | A2SG = ( 0/2 * (0) + 1/2 * ( H4N4E1 + H4N4F1E1 + H4N5E1 + H4N5F1E1 ) + 2/2 * ( H5N4L1 + H5N4E1 + H5N4F1L1 + H5N4F1E1 + H5N5E1 + H5N4L2 + H5N4E1L1 + H5N4F2E1 + H5N4E2 + H5N5F1E1 + H5N4F1L2 + H5N4F1E1L1 + H5N4F1E2 + H5N5E2 + H5N5F1E2 ) ) / ( H4N4E1 + H5N4L1 + H4N4F1E1 + H5N4E1 + H4N5E1 + H5N4F1L1 + H5N4F1E1 + H4N5F1E1 + H5N5E1 + H5N4L2 + H5N4E1L1 + H5N4F2E1 + H5N4E2 + H5N5F1E1 + H5N4F1L2 + H5N4F1E1L1 + H5N4F1E2 + H5N5E2 + H5N5F1E2 ) |
| A2F0S0G | Galactosylation per antenna within non-fucosylated, non-sialylated diantennary glycans | A2F0S0G = ( 0/2 * ( H3N5 ) + 1/2 * ( H4N4 + H4N5 ) + 2/2 * ( H5N4 + H5N5 ) ) / ( H4N4 + H3N5 + H5N4 + H4N5 + H5N5 ) |
| A2FS0G | Galactosylation per antenna within fucosylated non-sialylated diantennary glycans | A2FS0G = ( 0/2 * ( H3N4F1 + H3N5F1 ) + 1/2 * ( H4N4F1 + H4N5F1 ) + 2/2 * ( H5N4F1 + H5N5F1 ) ) / ( H3N4F1 + H4N4F1 + H3N5F1 + H5N4F1 + H4N5F1 + H5N5F1 ) |
| A2F0SG | Galactosylation per antenna within non-fucosylated sialylated diantennary glycans | A2F0SG = ( 0/2 * (0) + 1/2 * ( H4N4E1 + H4N5E1 ) + 2/2 * ( H5N4L1 + H5N4E1 + H5N5E1 + H5N4L2 + H5N4E1L1 + H5N4E2 + H5N5E2 ) ) / ( H4N4E1 + H5N4L1 + H5N4E1 + H4N5E1 + H5N5E1 + H5N4L2 + H5N4E1L1 + H5N4E2 + H5N5E2 ) |
| A2FSG | Galactosylation per antenna within fucosylated sialylated diantennary glycans | A2FSG = ( 0/2 * (0) + 1/2 * ( H4N4F1E1 + H4N5F1E1 ) + 2/2 * ( H5N4F1L1 + H5N4F1E1 + H5N4F2E1 + H5N5F1E1 + H5N4F1L2 + H5N4F1E1L1 + H5N4F1E2 + H5N5F1E2 ) ) / ( H4N4F1E1 + H5N4F1L1 + H5N4F1E1 + H4N5F1E1 + H5N4F2E1 + H5N5F1E1 + H5N4F1L2 + H5N4F1E1L1 + H5N4F1E2 + H5N5F1E2 ) |
| **Sialylation (S)** |  |  |
| CS | Sialylation per antenna within all complex glycans | CS = ( H3N3E1 + H3N3F1E1 + H4N3E1 + H4N3F1E1 + H4N4E1 + H5N4L1 + H4N4F1E1 + H5N4E1 + H4N5E1 + H5N4F1L1 + H5N4F1E1 + H4N5F1E1 + H5N5E1 + H5N4L2 + H5N4E1L1 + H5N4F2E1 + H5N4E2 + H5N5F1E1 + H6N5E1 + H5N4F1L2 + H4N6F1E1 + H5N4F1E1L1 + H5N4F1E2 + H6N5F1E1 + H5N5E2 + H6N5E1L1 + H5N5F1E2 + H6N5E2 + H6N5F1E1L1 + H6N5F1E2 + H6N5E1L2 + H6N5E2L1 + H6N5E3 + H6N5F1E1L2 + H6N5F1E2L1 + H6N5F1E3 + H7N6E1L2 + H7N6E2L2 ) / ( H3N3E1 + H3N4F1 + H4N4 + H3N5 + H3N3F1E1 + H4N3E1 + H4N4F1 + H5N4 + H3N5F1 + H4N5 + H4N3F1E1 + H5N4F1 + H4N4E1 + H4N5F1 + H5N5 + H5N4L1 + H4N4F1E1 + H5N4E1 + H5N5F1 + H4N5E1 + H6N5 + H5N4F1L1 + H5N4F1E1 + H4N5F1E1 + H5N5E1 + H5N4L2 + H5N4E1L1 + H5N4F2E1 + H5N4E2 + H5N5F1E1 + H6N5E1 + H5N4F1L2 + H4N6F1E1 + H5N4F1E1L1 + H5N4F1E2 + H6N5F1E1 + H5N5E2 + H6N5E1L1 + H5N5F1E2 + H6N5E2 + H6N5F1E1L1 + H6N5F1E2 + H6N5E1L2 + H6N5E2L1 + H6N5E3 + H6N5F1E1L2 + H6N5F1E2L1 + H6N5F1E3 + H7N6E1L2 + H7N6E2L2 ) |
| A2S | Sialylation per antenna within diantennary glycans | A2S = ( 0/2 * ( H3N4F1 + H4N4 + H3N5 + H4N4F1 + H5N4 + H3N5F1 + H4N5 + H5N4F1 + H4N5F1 + H5N5 + H5N5F1 ) + 1/2 * ( H4N4E1 + H5N4L1 + H4N4F1E1 + H5N4E1 + H4N5E1 + H5N4F1L1 + H5N4F1E1 + H4N5F1E1 + H5N5E1 + H5N4F2E1 + H5N5F1E1 ) + 2/2 * ( H5N4L2 + H5N4E1L1 + H5N4E2 + H5N4F1L2 + H5N4F1E1L1 + H5N4F1E2 + H5N5E2 + H5N5F1E2 ) ) / ( H3N4F1 + H4N4 + H3N5 + H4N4F1 + H5N4 + H3N5F1 + H4N5 + H5N4F1 + H4N4E1 + H4N5F1 + H5N5 + H5N4L1 + H4N4F1E1 + H5N4E1 + H5N5F1 + H4N5E1 + H5N4F1L1 + H5N4F1E1 + H4N5F1E1 + H5N5E1 + H5N4L2 + H5N4E1L1 + H5N4F2E1 + H5N4E2 + H5N5F1E1 + H5N4F1L2 + H5N4F1E1L1 + H5N4F1E2 + H5N5E2 + H5N5F1E2 ) |
| A3S | Sialylation per antenna within triantennary glycans | A3S = ( 0/3 * ( H6N5 ) + 1/3 * ( H6N5E1 + H6N5F1E1 ) + 2/3 * ( H6N5E1L1 + H6N5E2 + H6N5F1E1L1 + H6N5F1E2 ) + 3/3 * ( H6N5E1L2 + H6N5E2L1 + H6N5E3 + H6N5F1E1L2 + H6N5F1E2L1 + H6N5F1E3 ) ) / ( H6N5 + H6N5E1 + H6N5F1E1 + H6N5E1L1 + H6N5E2 + H6N5F1E1L1 + H6N5F1E2 + H6N5E1L2 + H6N5E2L1 + H6N5E3 + H6N5F1E1L2 + H6N5F1E2L1 + H6N5F1E3 ) |
| A4S | Sialylation per antenna within tetra-antennary glycans | A4S = ( 0/4 * (0) + 1/4 * ( H4N6F1E1 ) + 2/4 * (0) + 3/4 * ( H7N6E1L2 ) + 4/4 * ( H7N6E2L2 ) ) / ( H4N6F1E1 + H7N6E1L2 + H7N6E2L2 ) |
| A2F0S | Sialylation per antenna within non-fucosylated diantennary glycans | A2F0S = ( 0/2 * ( H4N4 + H3N5 + H5N4 + H4N5 + H5N5 ) + 1/2 * ( H4N4E1 + H5N4L1 + H5N4E1 + H4N5E1 + H5N5E1 ) + 2/2 * ( H5N4L2 + H5N4E1L1 + H5N4E2 + H5N5E2 ) ) / ( H4N4 + H3N5 + H5N4 + H4N5 + H4N4E1 + H5N5 + H5N4L1 + H5N4E1 + H4N5E1 + H5N5E1 + H5N4L2 + H5N4E1L1 + H5N4E2 + H5N5E2 ) |
| A3F0S | Sialylation per antenna within non-fucosylated triantennary glycans | A3F0S = ( 0/3 * ( H6N5 ) + 1/3 * ( H6N5E1 ) + 2/3 * ( H6N5E1L1 + H6N5E2 ) + 3/3 * ( H6N5E1L2 + H6N5E2L1 + H6N5E3 ) ) / ( H6N5 + H6N5E1 + H6N5E1L1 + H6N5E2 + H6N5E1L2 + H6N5E2L1 + H6N5E3 ) |
| A2FS | Sialylation per antenna within fucosylated diantennary glycans | A2FS = ( 0/2 * ( H3N4F1 + H4N4F1 + H3N5F1 + H5N4F1 + H4N5F1 + H5N5F1 ) + 1/2 * ( H4N4F1E1 + H5N4F1L1 + H5N4F1E1 + H4N5F1E1 + H5N4F2E1 + H5N5F1E1 ) + 2/2 * ( H5N4F1L2 + H5N4F1E1L1 + H5N4F1E2 + H5N5F1E2 ) ) / ( H3N4F1 + H4N4F1 + H3N5F1 + H5N4F1 + H4N5F1 + H4N4F1E1 + H5N5F1 + H5N4F1L1 + H5N4F1E1 + H4N5F1E1 + H5N4F2E1 + H5N5F1E1 + H5N4F1L2 + H5N4F1E1L1 + H5N4F1E2 + H5N5F1E2 ) |
| A3FS | Sialylation per antenna within fucosylated triantennary glycans | A3FS = ( 0/3 * (0) + 1/3 * ( H6N5F1E1 ) + 2/3 * ( H6N5F1E1L1 + H6N5F1E2 ) + 3/3 * ( H6N5F1E1L2 + H6N5F1E2L1 + H6N5F1E3 ) ) / ( H6N5F1E1 + H6N5F1E1L1 + H6N5F1E2 + H6N5F1E1L2 + H6N5F1E2L1 + H6N5F1E3 ) |
| A2GS | Sialylation per galactose within diantennary glycans | A2GS = ( ( 0/2 * ( H3N4F1 + H4N4 + H3N5 + H4N4F1 + H5N4 + H3N5F1 + H4N5 + H5N4F1 + H4N5F1 + H5N5 + H5N5F1 ) + 1/2 * ( H4N4E1 + H5N4L1 + H4N4F1E1 + H5N4E1 + H4N5E1 + H5N4F1L1 + H5N4F1E1 + H4N5F1E1 + H5N5E1 + H5N4F2E1 + H5N5F1E1 ) + 2/2 * ( H5N4L2 + H5N4E1L1 + H5N4E2 + H5N4F1L2 + H5N4F1E1L1 + H5N4F1E2 + H5N5E2 + H5N5F1E2 ) ) / ( H3N4F1 + H4N4 + H3N5 + H4N4F1 + H5N4 + H3N5F1 + H4N5 + H5N4F1 + H4N4E1 + H4N5F1 + H5N5 + H5N4L1 + H4N4F1E1 + H5N4E1 + H5N5F1 + H4N5E1 + H5N4F1L1 + H5N4F1E1 + H4N5F1E1 + H5N5E1 + H5N4L2 + H5N4E1L1 + H5N4F2E1 + H5N4E2 + H5N5F1E1 + H5N4F1L2 + H5N4F1E1L1 + H5N4F1E2 + H5N5E2 + H5N5F1E2 ) ) / ( ( 0/2 * ( H3N4F1 + H3N5 + H3N5F1 ) + 1/2 * ( H4N4 + H4N4F1 + H4N5 + H4N4E1 + H4N5F1 + H4N4F1E1 + H4N5E1 + H4N5F1E1 ) + 2/2 * ( H5N4 + H5N4F1 + H5N5 + H5N4L1 + H5N4E1 + H5N5F1 + H5N4F1L1 + H5N4F1E1 + H5N5E1 + H5N4L2 + H5N4E1L1 + H5N4F2E1 + H5N4E2 + H5N5F1E1 + H5N4F1L2 + H5N4F1E1L1 + H5N4F1E2 + H5N5E2 + H5N5F1E2 ) ) / ( H3N4F1 + H4N4 + H3N5 + H4N4F1 + H5N4 + H3N5F1 + H4N5 + H5N4F1 + H4N4E1 + H4N5F1 + H5N5 + H5N4L1 + H4N4F1E1 + H5N4E1 + H5N5F1 + H4N5E1 + H5N4F1L1 + H5N4F1E1 + H4N5F1E1 + H5N5E1 + H5N4L2 + H5N4E1L1 + H5N4F2E1 + H5N4E2 + H5N5F1E1 + H5N4F1L2 + H5N4F1E1L1 + H5N4F1E2 + H5N5E2 + H5N5F1E2 ) ) |
| A4GS | Sialylation per galactose within tetra-antennary glycans | A4GS = ( ( 0/4 * (0) + 1/4 * ( H4N6F1E1 ) + 2/4 * (0) + 3/4 * ( H7N6E1L2 ) + 4/4 * ( H7N6E2L2 ) ) / ( H4N6F1E1 + H7N6E1L2 + H7N6E2L2 ) ) / ( ( 0/4 * (0) + 1/4 * ( H4N6F1E1 ) + 2/4 * (0) + 3/4 * (0) + 4/4 * ( H7N6E1L2 + H7N6E2L2 ) ) / ( H4N6F1E1 + H7N6E1L2 + H7N6E2L2 ) ) |
| A2F0GS | Sialylation per galactose within non-fucosylated diantennary glycans | A2F0GS = ( ( 0/2 * ( H4N4 + H3N5 + H5N4 + H4N5 + H5N5 ) + 1/2 * ( H4N4E1 + H5N4L1 + H5N4E1 + H4N5E1 + H5N5E1 ) + 2/2 * ( H5N4L2 + H5N4E1L1 + H5N4E2 + H5N5E2 ) ) / ( H4N4 + H3N5 + H5N4 + H4N5 + H4N4E1 + H5N5 + H5N4L1 + H5N4E1 + H4N5E1 + H5N5E1 + H5N4L2 + H5N4E1L1 + H5N4E2 + H5N5E2 ) ) / ( ( 0/2 * ( H3N5 ) + 1/2 * ( H4N4 + H4N5 + H4N4E1 + H4N5E1 ) + 2/2 * ( H5N4 + H5N5 + H5N4L1 + H5N4E1 + H5N5E1 + H5N4L2 + H5N4E1L1 + H5N4E2 + H5N5E2 ) ) / ( H4N4 + H3N5 + H5N4 + H4N5 + H4N4E1 + H5N5 + H5N4L1 + H5N4E1 + H4N5E1 + H5N5E1 + H5N4L2 + H5N4E1L1 + H5N4E2 + H5N5E2 ) ) |
| A4F0GS | Sialylation per galactose within non-fucosylated tetra-antennary glycans | A4F0GS = ( ( 0/4 * (0) + 1/4 * (0) + 2/4 * (0) + 3/4 * ( H7N6E1L2 ) + 4/4 * ( H7N6E2L2 ) ) / ( H4N6F1E1 + H7N6E1L2 + H7N6E2L2 ) ) / ( ( 0/4 * (0) + 1/4 * (0) + 2/4 * (0) + 3/4 * (0) + 4/4 * ( H7N6E1L2 + H7N6E2L2 ) ) / ( H4N6F1E1 + H7N6E1L2 + H7N6E2L2 ) ) |
| A2FGS | Sialylation per galactose within fucosylated diantennary glycans | A2FGS = ( ( 0/2 * ( H3N4F1 + H4N4F1 + H3N5F1 + H5N4F1 + H4N5F1 + H5N5F1 ) + 1/2 * ( H4N4F1E1 + H5N4F1L1 + H5N4F1E1 + H4N5F1E1 + H5N4F2E1 + H5N5F1E1 ) + 2/2 * ( H5N4F1L2 + H5N4F1E1L1 + H5N4F1E2 + H5N5F1E2 ) ) / ( H3N4F1 + H4N4F1 + H3N5F1 + H5N4F1 + H4N5F1 + H4N4F1E1 + H5N5F1 + H5N4F1L1 + H5N4F1E1 + H4N5F1E1 + H5N4F2E1 + H5N5F1E1 + H5N4F1L2 + H5N4F1E1L1 + H5N4F1E2 + H5N5F1E2 ) ) / ( ( 0/2 * ( H3N4F1 + H3N5F1 ) + 1/2 * ( H4N4F1 + H4N5F1 + H4N4F1E1 + H4N5F1E1 ) + 2/2 * ( H5N4F1 + H5N5F1 + H5N4F1L1 + H5N4F1E1 + H5N4F2E1 + H5N5F1E1 + H5N4F1L2 + H5N4F1E1L1 + H5N4F1E2 + H5N5F1E2 ) ) / ( H3N4F1 + H4N4F1 + H3N5F1 + H5N4F1 + H4N5F1 + H4N4F1E1 + H5N5F1 + H5N4F1L1 + H5N4F1E1 + H4N5F1E1 + H5N4F2E1 + H5N5F1E1 + H5N4F1L2 + H5N4F1E1L1 + H5N4F1E2 + H5N5F1E2 ) ) |
| **α2,3-linked sialylation (L)** |  |  |
| A2L | α2,3-sialylation per antenna within diantennary glycans | A2L = ( 0/2 * ( H3N4F1 + H4N4 + H3N5 + H4N4F1 + H5N4 + H3N5F1 + H4N5 + H5N4F1 + H4N4E1 + H4N5F1 + H5N5 + H4N4F1E1 + H5N4E1 + H5N5F1 + H4N5E1 + H5N4F1E1 + H4N5F1E1 + H5N5E1 + H5N4F2E1 + H5N4E2 + H5N5F1E1 + H5N4F1E2 + H5N5E2 + H5N5F1E2 ) + 1/2 * ( H5N4L1 + H5N4F1L1 + H5N4E1L1 + H5N4F1E1L1 ) + 2/2 * ( H5N4L2 + H5N4F1L2 ) ) / ( H3N4F1 + H4N4 + H3N5 + H4N4F1 + H5N4 + H3N5F1 + H4N5 + H5N4F1 + H4N4E1 + H4N5F1 + H5N5 + H5N4L1 + H4N4F1E1 + H5N4E1 + H5N5F1 + H4N5E1 + H5N4F1L1 + H5N4F1E1 + H4N5F1E1 + H5N5E1 + H5N4L2 + H5N4E1L1 + H5N4F2E1 + H5N4E2 + H5N5F1E1 + H5N4F1L2 + H5N4F1E1L1 + H5N4F1E2 + H5N5E2 + H5N5F1E2 ) |
| A3L | α2,3-sialylation per antenna within triantennary glycans | A3L = ( 0/3 * ( H6N5 + H6N5E1 + H6N5F1E1 + H6N5E2 + H6N5F1E2 + H6N5E3 + H6N5F1E3 ) + 1/3 * ( H6N5E1L1 + H6N5F1E1L1 + H6N5E2L1 + H6N5F1E2L1 ) + 2/3 * ( H6N5E1L2 + H6N5F1E1L2 ) + 3/3 * (0) ) / ( H6N5 + H6N5E1 + H6N5F1E1 + H6N5E1L1 + H6N5E2 + H6N5F1E1L1 + H6N5F1E2 + H6N5E1L2 + H6N5E2L1 + H6N5E3 + H6N5F1E1L2 + H6N5F1E2L1 + H6N5F1E3 ) |
| A4L | α2,3-sialylation per antenna within tetra-antennary glycans | A4L = ( 0/4 * ( H4N6F1E1 ) + 1/4 * (0) + 2/4 * ( H7N6E1L2 + H7N6E2L2 ) + 3/4 * (0) + 4/4 * (0) ) / ( H4N6F1E1 + H7N6E1L2 + H7N6E2L2 ) |
| A2F0L | α2,3-sialylation per antenna within non-fucosylated diantennary glycans | A2F0L = ( 0/2 * ( H4N4 + H3N5 + H5N4 + H4N5 + H4N4E1 + H5N5 + H5N4E1 + H4N5E1 + H5N5E1 + H5N4E2 + H5N5E2 ) + 1/2 * ( H5N4L1 + H5N4E1L1 ) + 2/2 * ( H5N4L2 ) ) / ( H4N4 + H3N5 + H5N4 + H4N5 + H4N4E1 + H5N5 + H5N4L1 + H5N4E1 + H4N5E1 + H5N5E1 + H5N4L2 + H5N4E1L1 + H5N4E2 + H5N5E2 ) |
| A3F0L | α2,3-sialylation per antenna within non-fucosylated triantennary glycans | A3F0L = ( 0/3 * ( H6N5 + H6N5E1 + H6N5E2 + H6N5E3 ) + 1/3 * ( H6N5E1L1 + H6N5E2L1 ) + 2/3 * ( H6N5E1L2 ) + 3/3 * (0) ) / ( H6N5 + H6N5E1 + H6N5E1L1 + H6N5E2 + H6N5E1L2 + H6N5E2L1 + H6N5E3 ) |
| A2FL | α2,3-sialylation per antenna within fucosylated diantennary glycans | A2FL = ( 0/2 * ( H3N4F1 + H4N4F1 + H3N5F1 + H5N4F1 + H4N5F1 + H4N4F1E1 + H5N5F1 + H5N4F1E1 + H4N5F1E1 + H5N4F2E1 + H5N5F1E1 + H5N4F1E2 + H5N5F1E2 ) + 1/2 * ( H5N4F1L1 + H5N4F1E1L1 ) + 2/2 * ( H5N4F1L2 ) ) / ( H3N4F1 + H4N4F1 + H3N5F1 + H5N4F1 + H4N5F1 + H4N4F1E1 + H5N5F1 + H5N4F1L1 + H5N4F1E1 + H4N5F1E1 + H5N4F2E1 + H5N5F1E1 + H5N4F1L2 + H5N4F1E1L1 + H5N4F1E2 + H5N5F1E2 ) |
| A3FL | α2,3-sialylation per antenna within fucosylated triantennary glycans | A3FL = ( 0/3 * ( H6N5F1E1 + H6N5F1E2 + H6N5F1E3 ) + 1/3 * ( H6N5F1E1L1 + H6N5F1E2L1 ) + 2/3 * ( H6N5F1E1L2 ) + 3/3 * (0) ) / ( H6N5F1E1 + H6N5F1E1L1 + H6N5F1E2 + H6N5F1E1L2 + H6N5F1E2L1 + H6N5F1E3 ) |
| A2GL | α2,3-sialylation per galactose within diantennary glycans | A2GL = ( ( 0/2 * ( H3N4F1 + H4N4 + H3N5 + H4N4F1 + H5N4 + H3N5F1 + H4N5 + H5N4F1 + H4N4E1 + H4N5F1 + H5N5 + H4N4F1E1 + H5N4E1 + H5N5F1 + H4N5E1 + H5N4F1E1 + H4N5F1E1 + H5N5E1 + H5N4F2E1 + H5N4E2 + H5N5F1E1 + H5N4F1E2 + H5N5E2 + H5N5F1E2 ) + 1/2 * ( H5N4L1 + H5N4F1L1 + H5N4E1L1 + H5N4F1E1L1 ) + 2/2 * ( H5N4L2 + H5N4F1L2 ) ) / ( H3N4F1 + H4N4 + H3N5 + H4N4F1 + H5N4 + H3N5F1 + H4N5 + H5N4F1 + H4N4E1 + H4N5F1 + H5N5 + H5N4L1 + H4N4F1E1 + H5N4E1 + H5N5F1 + H4N5E1 + H5N4F1L1 + H5N4F1E1 + H4N5F1E1 + H5N5E1 + H5N4L2 + H5N4E1L1 + H5N4F2E1 + H5N4E2 + H5N5F1E1 + H5N4F1L2 + H5N4F1E1L1 + H5N4F1E2 + H5N5E2 + H5N5F1E2 ) ) / ( ( 0/2 * ( H3N4F1 + H3N5 + H3N5F1 ) + 1/2 * ( H4N4 + H4N4F1 + H4N5 + H4N4E1 + H4N5F1 + H4N4F1E1 + H4N5E1 + H4N5F1E1 ) + 2/2 * ( H5N4 + H5N4F1 + H5N5 + H5N4L1 + H5N4E1 + H5N5F1 + H5N4F1L1 + H5N4F1E1 + H5N5E1 + H5N4L2 + H5N4E1L1 + H5N4F2E1 + H5N4E2 + H5N5F1E1 + H5N4F1L2 + H5N4F1E1L1 + H5N4F1E2 + H5N5E2 + H5N5F1E2 ) ) / ( H3N4F1 + H4N4 + H3N5 + H4N4F1 + H5N4 + H3N5F1 + H4N5 + H5N4F1 + H4N4E1 + H4N5F1 + H5N5 + H5N4L1 + H4N4F1E1 + H5N4E1 + H5N5F1 + H4N5E1 + H5N4F1L1 + H5N4F1E1 + H4N5F1E1 + H5N5E1 + H5N4L2 + H5N4E1L1 + H5N4F2E1 + H5N4E2 + H5N5F1E1 + H5N4F1L2 + H5N4F1E1L1 + H5N4F1E2 + H5N5E2 + H5N5F1E2 ) ) |
| A2F0GL | α2,3-sialylation per galactose within non-fucosylated diantennary glycans | A2F0GL = ( ( 0/2 * ( H4N4 + H3N5 + H5N4 + H4N5 + H4N4E1 + H5N5 + H5N4E1 + H4N5E1 + H5N5E1 + H5N4E2 + H5N5E2 ) + 1/2 * ( H5N4L1 + H5N4E1L1 ) + 2/2 * ( H5N4L2 ) ) / ( H4N4 + H3N5 + H5N4 + H4N5 + H4N4E1 + H5N5 + H5N4L1 + H5N4E1 + H4N5E1 + H5N5E1 + H5N4L2 + H5N4E1L1 + H5N4E2 + H5N5E2 ) ) / ( ( 0/2 * ( H3N5 ) + 1/2 * ( H4N4 + H4N5 + H4N4E1 + H4N5E1 ) + 2/2 * ( H5N4 + H5N5 + H5N4L1 + H5N4E1 + H5N5E1 + H5N4L2 + H5N4E1L1 + H5N4E2 + H5N5E2 ) ) / ( H4N4 + H3N5 + H5N4 + H4N5 + H4N4E1 + H5N5 + H5N4L1 + H5N4E1 + H4N5E1 + H5N5E1 + H5N4L2 + H5N4E1L1 + H5N4E2 + H5N5E2 ) ) |
| A2FGL | α2,3-sialylation per galactose within fucosylated diantennary glycans | A2FGL = ( ( 0/2 * ( H3N4F1 + H4N4F1 + H3N5F1 + H5N4F1 + H4N5F1 + H4N4F1E1 + H5N5F1 + H5N4F1E1 + H4N5F1E1 + H5N4F2E1 + H5N5F1E1 + H5N4F1E2 + H5N5F1E2 ) + 1/2 * ( H5N4F1L1 + H5N4F1E1L1 ) + 2/2 * ( H5N4F1L2 ) ) / ( H3N4F1 + H4N4F1 + H3N5F1 + H5N4F1 + H4N5F1 + H4N4F1E1 + H5N5F1 + H5N4F1L1 + H5N4F1E1 + H4N5F1E1 + H5N4F2E1 + H5N5F1E1 + H5N4F1L2 + H5N4F1E1L1 + H5N4F1E2 + H5N5F1E2 ) ) / ( ( 0/2 * ( H3N4F1 + H3N5F1 ) + 1/2 * ( H4N4F1 + H4N5F1 + H4N4F1E1 + H4N5F1E1 ) + 2/2 * ( H5N4F1 + H5N5F1 + H5N4F1L1 + H5N4F1E1 + H5N4F2E1 + H5N5F1E1 + H5N4F1L2 + H5N4F1E1L1 + H5N4F1E2 + H5N5F1E2 ) ) / ( H3N4F1 + H4N4F1 + H3N5F1 + H5N4F1 + H4N5F1 + H4N4F1E1 + H5N5F1 + H5N4F1L1 + H5N4F1E1 + H4N5F1E1 + H5N4F2E1 + H5N5F1E1 + H5N4F1L2 + H5N4F1E1L1 + H5N4F1E2 + H5N5F1E2 ) ) |
| **α2,6-linked sialylation (E)** |  |  |
| A2E | α2,6-sialylation per antenna within diantennary glycans | A2E = ( 0/2 * ( H3N4F1 + H4N4 + H3N5 + H4N4F1 + H5N4 + H3N5F1 + H4N5 + H5N4F1 + H4N5F1 + H5N5 + H5N4L1 + H5N5F1 + H5N4F1L1 + H5N4L2 + H5N4F1L2 ) + 1/2 * ( H4N4E1 + H4N4F1E1 + H5N4E1 + H4N5E1 + H5N4F1E1 + H4N5F1E1 + H5N5E1 + H5N4E1L1 + H5N4F2E1 + H5N5F1E1 + H5N4F1E1L1 ) + 2/2 * ( H5N4E2 + H5N4F1E2 + H5N5E2 + H5N5F1E2 ) ) / ( H3N4F1 + H4N4 + H3N5 + H4N4F1 + H5N4 + H3N5F1 + H4N5 + H5N4F1 + H4N4E1 + H4N5F1 + H5N5 + H5N4L1 + H4N4F1E1 + H5N4E1 + H5N5F1 + H4N5E1 + H5N4F1L1 + H5N4F1E1 + H4N5F1E1 + H5N5E1 + H5N4L2 + H5N4E1L1 + H5N4F2E1 + H5N4E2 + H5N5F1E1 + H5N4F1L2 + H5N4F1E1L1 + H5N4F1E2 + H5N5E2 + H5N5F1E2 ) |
| A3E | α2,6-sialylation per antenna within triantennary glycans | A3E = ( 0/3 * ( H6N5 ) + 1/3 * ( H6N5E1 + H6N5F1E1 + H6N5E1L1 + H6N5F1E1L1 + H6N5E1L2 + H6N5F1E1L2 ) + 2/3 * ( H6N5E2 + H6N5F1E2 + H6N5E2L1 + H6N5F1E2L1 ) + 3/3 * ( H6N5E3 + H6N5F1E3 ) ) / ( H6N5 + H6N5E1 + H6N5F1E1 + H6N5E1L1 + H6N5E2 + H6N5F1E1L1 + H6N5F1E2 + H6N5E1L2 + H6N5E2L1 + H6N5E3 + H6N5F1E1L2 + H6N5F1E2L1 + H6N5F1E3 ) |
| A4E | α2,6-sialylation per antenna within tetra-antennary glycans | A4E = ( 0/4 * (0) + 1/4 * ( H4N6F1E1 + H7N6E1L2 ) + 2/4 * ( H7N6E2L2 ) + 3/4 * (0) + 4/4 * (0) ) / ( H4N6F1E1 + H7N6E1L2 + H7N6E2L2 ) |
| A2F0E | α2,6-sialylation per antenna within non-fucosylated diantennary glycans | A2F0E = ( 0/2 * ( H4N4 + H3N5 + H5N4 + H4N5 + H5N5 + H5N4L1 + H5N4L2 ) + 1/2 * ( H4N4E1 + H5N4E1 + H4N5E1 + H5N5E1 + H5N4E1L1 ) + 2/2 * ( H5N4E2 + H5N5E2 ) ) / ( H4N4 + H3N5 + H5N4 + H4N5 + H4N4E1 + H5N5 + H5N4L1 + H5N4E1 + H4N5E1 + H5N5E1 + H5N4L2 + H5N4E1L1 + H5N4E2 + H5N5E2 ) |
| A3F0E | α2,6-sialylation per antenna within non-fucosylated triantennary glycans | A3F0E = ( 0/3 * ( H6N5 ) + 1/3 * ( H6N5E1 + H6N5E1L1 + H6N5E1L2 ) + 2/3 * ( H6N5E2 + H6N5E2L1 ) + 3/3 * ( H6N5E3 ) ) / ( H6N5 + H6N5E1 + H6N5E1L1 + H6N5E2 + H6N5E1L2 + H6N5E2L1 + H6N5E3 ) |
| A4F0E | α2,6-sialylation per antenna within non-fucosylated tetra-antennary glycans | A4F0E = ( 0/4 * (0) + 1/4 * ( H7N6E1L2 ) + 2/4 * ( H7N6E2L2 ) + 3/4 * (0) + 4/4 * (0) ) / ( H4N6F1E1 + H7N6E1L2 + H7N6E2L2 ) |
| A2FE | α2,6-sialylation per antenna within fucosylated diantennary glycans | A2FE = ( 0/2 * ( H3N4F1 + H4N4F1 + H3N5F1 + H5N4F1 + H4N5F1 + H5N5F1 + H5N4F1L1 + H5N4F1L2 ) + 1/2 * ( H4N4F1E1 + H5N4F1E1 + H4N5F1E1 + H5N4F2E1 + H5N5F1E1 + H5N4F1E1L1 ) + 2/2 * ( H5N4F1E2 + H5N5F1E2 ) ) / ( H3N4F1 + H4N4F1 + H3N5F1 + H5N4F1 + H4N5F1 + H4N4F1E1 + H5N5F1 + H5N4F1L1 + H5N4F1E1 + H4N5F1E1 + H5N4F2E1 + H5N5F1E1 + H5N4F1L2 + H5N4F1E1L1 + H5N4F1E2 + H5N5F1E2 ) |
| A3FE | α2,6-sialylation per antenna within fucosylated triantennary glycans | A3FE = ( 0/3 * (0) + 1/3 * ( H6N5F1E1 + H6N5F1E1L1 + H6N5F1E1L2 ) + 2/3 * ( H6N5F1E2 + H6N5F1E2L1 ) + 3/3 * ( H6N5F1E3 ) ) / ( H6N5F1E1 + H6N5F1E1L1 + H6N5F1E2 + H6N5F1E1L2 + H6N5F1E2L1 + H6N5F1E3 ) |
| A4FE | α2,6-sialylation per antenna within fucosylated tetra-antennary glycans | A4FE = ( 0/4 * (0) + 1/4 * ( H4N6F1E1 ) + 2/4 * (0) + 3/4 * (0) + 4/4 * (0) ) / ( H4N6F1E1 + H7N6E1L2 + H7N6E2L2 ) |
| A2GE | α2,6-sialylation per galactose within diantennary glycans | A2GE = ( ( 0/2 * ( H3N4F1 + H4N4 + H3N5 + H4N4F1 + H5N4 + H3N5F1 + H4N5 + H5N4F1 + H4N5F1 + H5N5 + H5N4L1 + H5N5F1 + H5N4F1L1 + H5N4L2 + H5N4F1L2 ) + 1/2 * ( H4N4E1 + H4N4F1E1 + H5N4E1 + H4N5E1 + H5N4F1E1 + H4N5F1E1 + H5N5E1 + H5N4E1L1 + H5N4F2E1 + H5N5F1E1 + H5N4F1E1L1 ) + 2/2 * ( H5N4E2 + H5N4F1E2 + H5N5E2 + H5N5F1E2 ) ) / ( H3N4F1 + H4N4 + H3N5 + H4N4F1 + H5N4 + H3N5F1 + H4N5 + H5N4F1 + H4N4E1 + H4N5F1 + H5N5 + H5N4L1 + H4N4F1E1 + H5N4E1 + H5N5F1 + H4N5E1 + H5N4F1L1 + H5N4F1E1 + H4N5F1E1 + H5N5E1 + H5N4L2 + H5N4E1L1 + H5N4F2E1 + H5N4E2 + H5N5F1E1 + H5N4F1L2 + H5N4F1E1L1 + H5N4F1E2 + H5N5E2 + H5N5F1E2 ) ) / ( ( 0/2 * ( H3N4F1 + H3N5 + H3N5F1 ) + 1/2 * ( H4N4 + H4N4F1 + H4N5 + H4N4E1 + H4N5F1 + H4N4F1E1 + H4N5E1 + H4N5F1E1 ) + 2/2 * ( H5N4 + H5N4F1 + H5N5 + H5N4L1 + H5N4E1 + H5N5F1 + H5N4F1L1 + H5N4F1E1 + H5N5E1 + H5N4L2 + H5N4E1L1 + H5N4F2E1 + H5N4E2 + H5N5F1E1 + H5N4F1L2 + H5N4F1E1L1 + H5N4F1E2 + H5N5E2 + H5N5F1E2 ) ) / ( H3N4F1 + H4N4 + H3N5 + H4N4F1 + H5N4 + H3N5F1 + H4N5 + H5N4F1 + H4N4E1 + H4N5F1 + H5N5 + H5N4L1 + H4N4F1E1 + H5N4E1 + H5N5F1 + H4N5E1 + H5N4F1L1 + H5N4F1E1 + H4N5F1E1 + H5N5E1 + H5N4L2 + H5N4E1L1 + H5N4F2E1 + H5N4E2 + H5N5F1E1 + H5N4F1L2 + H5N4F1E1L1 + H5N4F1E2 + H5N5E2 + H5N5F1E2 ) ) |
| A4GE | α2,6-sialylation per galactose within tetra-antennary glycans | A4GE = ( ( 0/4 * (0) + 1/4 * ( H4N6F1E1 + H7N6E1L2 ) + 2/4 * ( H7N6E2L2 ) + 3/4 * (0) + 4/4 * (0) ) / ( H4N6F1E1 + H7N6E1L2 + H7N6E2L2 ) ) / ( ( 0/4 * (0) + 1/4 * ( H4N6F1E1 ) + 2/4 * (0) + 3/4 * (0) + 4/4 * ( H7N6E1L2 + H7N6E2L2 ) ) / ( H4N6F1E1 + H7N6E1L2 + H7N6E2L2 ) ) |
| A2F0GE | α2,6-sialylation per galactose within non-fucosylated diantennary glycans | A2F0GE = ( ( 0/2 * ( H4N4 + H3N5 + H5N4 + H4N5 + H5N5 + H5N4L1 + H5N4L2 ) + 1/2 * ( H4N4E1 + H5N4E1 + H4N5E1 + H5N5E1 + H5N4E1L1 ) + 2/2 * ( H5N4E2 + H5N5E2 ) ) / ( H4N4 + H3N5 + H5N4 + H4N5 + H4N4E1 + H5N5 + H5N4L1 + H5N4E1 + H4N5E1 + H5N5E1 + H5N4L2 + H5N4E1L1 + H5N4E2 + H5N5E2 ) ) / ( ( 0/2 * ( H3N5 ) + 1/2 * ( H4N4 + H4N5 + H4N4E1 + H4N5E1 ) + 2/2 * ( H5N4 + H5N5 + H5N4L1 + H5N4E1 + H5N5E1 + H5N4L2 + H5N4E1L1 + H5N4E2 + H5N5E2 ) ) / ( H4N4 + H3N5 + H5N4 + H4N5 + H4N4E1 + H5N5 + H5N4L1 + H5N4E1 + H4N5E1 + H5N5E1 + H5N4L2 + H5N4E1L1 + H5N4E2 + H5N5E2 ) ) |
| A4F0GE | α2,6-sialylation per galactose within non-fucosylated tetra-antennary glycans | A4F0GE = ( ( 0/4 * (0) + 1/4 * ( H7N6E1L2 ) + 2/4 * ( H7N6E2L2 ) + 3/4 * (0) + 4/4 * (0) ) / ( H4N6F1E1 + H7N6E1L2 + H7N6E2L2 ) ) / ( ( 0/4 * (0) + 1/4 * (0) + 2/4 * (0) + 3/4 * (0) + 4/4 * ( H7N6E1L2 + H7N6E2L2 ) ) / ( H4N6F1E1 + H7N6E1L2 + H7N6E2L2 ) ) |
| A2FGE | α2,6-sialylation per galactose within fucosylated diantennary glycans | A2FGE = ( ( 0/2 * ( H3N4F1 + H4N4F1 + H3N5F1 + H5N4F1 + H4N5F1 + H5N5F1 + H5N4F1L1 + H5N4F1L2 ) + 1/2 * ( H4N4F1E1 + H5N4F1E1 + H4N5F1E1 + H5N4F2E1 + H5N5F1E1 + H5N4F1E1L1 ) + 2/2 * ( H5N4F1E2 + H5N5F1E2 ) ) / ( H3N4F1 + H4N4F1 + H3N5F1 + H5N4F1 + H4N5F1 + H4N4F1E1 + H5N5F1 + H5N4F1L1 + H5N4F1E1 + H4N5F1E1 + H5N4F2E1 + H5N5F1E1 + H5N4F1L2 + H5N4F1E1L1 + H5N4F1E2 + H5N5F1E2 ) ) / ( ( 0/2 * ( H3N4F1 + H3N5F1 ) + 1/2 * ( H4N4F1 + H4N5F1 + H4N4F1E1 + H4N5F1E1 ) + 2/2 * ( H5N4F1 + H5N5F1 + H5N4F1L1 + H5N4F1E1 + H5N4F2E1 + H5N5F1E1 + H5N4F1L2 + H5N4F1E1L1 + H5N4F1E2 + H5N5F1E2 ) ) / ( H3N4F1 + H4N4F1 + H3N5F1 + H5N4F1 + H4N5F1 + H4N4F1E1 + H5N5F1 + H5N4F1L1 + H5N4F1E1 + H4N5F1E1 + H5N4F2E1 + H5N5F1E1 + H5N4F1L2 + H5N4F1E1L1 + H5N4F1E2 + H5N5F1E2 ) ) |
| IgG-specific |  |  |
| TA2FS0 | Fucosylated, non-sialylated diantennary species within total glycans. Mostly derived from IgG (Clerc et al. 2015). | TA2FS0 = ( H3N4F1 + H4N4F1 + H3N5F1 + H5N4F1 + H4N5F1 + H5N5F1 ) |

**Supplemental Table S4. Data quality control.** Data quality was checked by measuring a standard sample, assessed by calculating the mean, SD (standard deviation), and relative SD (RSD) for all glycan traits.

| **Directly detected glycan traits** | **Average (Relative abundance)** | **SD** | **relative SD of directly traits** |  | **Derived glycan traits** | **Average**  **(Relative abundance)** | **SD** | **relative SD of derived traits**  **(mean is 2.32% for all)** |
| --- | --- | --- | --- | --- | --- | --- | --- | --- |
| H5N4E2 | 26.93477026 | 0.350020417 | 1.30% |  | TM | 0.031992037 | 0.002976242 | 9.30% |
| H4N4F1 | 7.18534783 | 0.259269266 | 3.61% |  | THy | 0.012638253 | 0.000379524 | 3.00% |
| H5N4E1 | 6.125873002 | 0.100733182 | 1.64% |  | MHy | 2.5202089 | 0.254981746 | 10.12% |
| H5N4F1E1 | 4.937331041 | 0.076507385 | 1.55% |  | MM | 6.504137265 | 0.025116181 | 0.39% |
| H5N5F1E1 | 4.721791354 | 0.083056537 | 1.76% |  | CA1 | 0.012087122 | 0.000441135 | 3.65% |
| H5N4F1 | 4.506023286 | 0.152445892 | 3.38% |  | CA2 | 0.877411193 | 0.005506723 | 0.63% |
| H3N4F1 | 4.292239765 | 0.170712072 | 3.98% |  | CA3 | 0.093265535 | 0.003284433 | 3.52% |
| H6N5E2L1 | 3.712385053 | 0.180081274 | 4.85% |  | CA4 | 0.018798088 | 0.001629718 | 8.67% |
| H5N4E1L1 | 3.601221721 | 0.087387772 | 2.43% |  | CF | 0.462103649 | 0.003811235 | 0.82% |
| H5N4F1E2 | 3.557080187 | 0.131045402 | 3.68% |  | CFa | 0.000898591 | 8.30149E-05 | 9.24% |
| H5N5F1E2 | 3.543331124 | 0.131677275 | 3.72% |  | CB | 0.173692382 | 0.001779122 | 1.02% |
| H4N5F1 | 2.347423147 | 0.046656861 | 1.99% |  | CG | 0.939921257 | 0.001855721 | 0.20% |
| H4N5F1E1 | 1.415330488 | 0.048636165 | 3.44% |  | CS | 0.761110451 | 0.006855198 | 0.90% |
| H4N5E1 | 1.338176545 | 0.091143569 | 6.81% |  | TA2FS0 | 21.03822697 | 0.659631464 | 3.14% |
| H3N5F1 | 1.326820024 | 0.03786336 | 2.85% |  | A1F | 0.561157363 | 0.008339431 | 1.49% |
| H6N5E3 | 1.260976452 | 0.055996908 | 4.44% |  | A2F | 0.495000607 | 0.002397415 | 0.48% |
| H6N2 | 1.222562662 | 0.127466402 | 10.43% |  | A3F | 0.186884985 | 0.007175971 | 3.84% |
| H5N5F1 | 1.146674669 | 0.042677642 | 3.72% |  | A2Fa | 0.001049083 | 9.70463E-05 | 9.25% |
| H5N4F1E1L1 | 0.864991719 | 0.040378059 | 4.67% |  | A2S0F | 0.911704586 | 0.005086052 | 0.56% |
| H5N2 | 0.856029231 | 0.071409695 | 8.34% |  | A2L0F | 0.513464125 | 0.002448121 | 0.48% |
| H4N4 | 0.836785892 | 0.073337336 | 8.76% |  | A3L0F | 0.274960775 | 0.010809709 | 3.93% |
| H5N4L2 | 0.821084535 | 0.030746856 | 3.74% |  | A2E0F | 0.867688748 | 0.003566347 | 0.41% |
| H5N5E1 | 0.801688581 | 0.050312268 | 6.28% |  | A2SF | 0.344180108 | 0.003282323 | 0.95% |
| H4N4E1 | 0.759042427 | 0.030758182 | 4.05% |  | A2LF | 0.301433984 | 0.002943476 | 0.98% |
| H8N2 | 0.72845488 | 0.075634131 | 10.38% |  | A3LF | 0.142027323 | 0.004145118 | 2.92% |
| H4N4F1E1 | 0.696810506 | 0.010014432 | 1.44% |  | A2EF | 0.337533665 | 0.003435258 | 1.02% |
| H6N5E1L2 | 0.678475895 | 0.034031428 | 5.02% |  | A3EF | 0.190477106 | 0.007347529 | 3.86% |
| H4N6F1E1 | 0.651852932 | 0.052793753 | 8.10% |  | A2B | 0.200940484 | 0.002893568 | 1.44% |
| H6N5E1L1 | 0.651582653 | 0.028383542 | 4.36% |  | A2F0B | 0.064043541 | 0.003657926 | 5.71% |
| H5N4F1L1 | 0.630984621 | 0.023305104 | 3.69% |  | A2FB | 0.343070463 | 0.004689681 | 1.37% |
| H5N4F1L2 | 0.598852351 | 0.029873785 | 4.99% |  | A2S0B | 0.241052399 | 0.003802946 | 1.58% |
| H5N3E1 | 0.581264682 | 0.016441316 | 2.83% |  | A2SB | 0.185121848 | 0.002986354 | 1.61% |
| H6N5F1E2L1 | 0.561541013 | 0.022275429 | 3.97% |  | A2F0S0B | 0.339507721 | 0.012798766 | 3.77% |
| H5N4L1 | 0.5440475 | 0.009547378 | 1.75% |  | A2F0SB | 0.050680871 | 0.002833589 | 5.59% |
| H7N6E2L2 | 0.513775168 | 0.057357281 | 11.16% |  | A2FS0B | 0.232982011 | 0.003130926 | 1.34% |
| H5N4 | 0.45275658 | 0.035357535 | 7.81% |  | A2FSB | 0.452331903 | 0.00395847 | 0.88% |
| H4N3F1E1 | 0.399168943 | 0.015964612 | 4.00% |  | A2G | 0.844068185 | 0.003736552 | 0.44% |
| H9N2 | 0.380496954 | 0.04541578 | 11.94% |  | A4G | 0.649881612 | 0.029181327 | 4.49% |
| H4N3E1 | 0.377071723 | 0.018562632 | 4.92% |  | A2F0G | 0.957467788 | 0.002562384 | 0.27% |
| H6N5F1E2 | 0.335594599 | 0.034266027 | 10.21% |  | A2FG | 0.728746572 | 0.006847771 | 0.94% |
| H6N5F1E1 | 0.327562058 | 0.010740913 | 3.28% |  | A2S0G | 0.514486213 | 0.002866019 | 0.56% |
| H6N5E2 | 0.325544324 | 0.015466917 | 4.75% |  | A2SG | 0.964935417 | 0.00038617 | 0.04% |
| H6N3 | 0.323176552 | 0.16271505 | 50.35% |  | A2F0S0G | 0.605675417 | 0.01040105 | 1.72% |
| H6N3E1 | 0.316114477 | 0.010060319 | 3.18% |  | A2FS0G | 0.504282103 | 0.003275132 | 0.65% |
| H6N5E1 | 0.305547237 | 0.011661548 | 3.82% |  | A2F0SG | 0.977557091 | 0.000696313 | 0.07% |
| H4N5 | 0.270913334 | 0.02092143 | 7.72% |  | A2FSG | 0.953616318 | 0.000630772 | 0.07% |
| H7N6E1L2 | 0.269348905 | 0.03609665 | 13.40% |  | A2S | 0.604600634 | 0.006007785 | 0.99% |
| H3N3F1E1 | 0.244638531 | 0.043772119 | 17.89% |  | A3S | 0.868658712 | 0.004525407 | 0.52% |
| H6N5 | 0.218964467 | 0.014772664 | 6.75% |  | A4S | 0.588673378 | 0.023189733 | 3.94% |
| H5N5E2 | 0.206890351 | 0.0112341 | 5.43% |  | A2F0S | 0.84283588 | 0.004645138 | 0.55% |
| H5N5 | 0.197872981 | 0.011023735 | 5.57% |  | A3F0S | 0.892865405 | 0.00430671 | 0.48% |
| H3N5 | 0.179652069 | 0.021163087 | 11.78% |  | A2FS | 0.349570239 | 0.010261351 | 2.94% |
| H6N5F1E1L1 | 0.171386457 | 0.012194389 | 7.12% |  | A3FS | 0.767224034 | 0.002823234 | 0.37% |
| H5N3 | 0.166903876 | 0.013359801 | 8.00% |  | A2L | 0.050852591 | 0.001269338 | 2.50% |
| H6N5F1E1L2 | 0.135987581 | 0.006139978 | 4.52% |  | A3L | 0.257291465 | 0.002702737 | 1.05% |
| H6N5F1E3 | 0.128879929 | 0.005354994 | 4.16% |  | A4L | 0.237994365 | 0.015676732 | 6.59% |
| H3N3E1 | 0.127840804 | 0.012204107 | 9.55% |  | A2F0L | 0.06837301 | 0.001102852 | 1.61% |
| H4N3F1 | 0.10479834 | 0.009626694 | 9.19% |  | A3F0L | 0.257885232 | 0.001926787 | 0.75% |
| H5N4F2E1 | 0.086555066 | 0.008083072 | 9.34% |  | A2FL | 0.033732261 | 0.000962249 | 2.85% |
|  |  |  |  |  | A3FL | 0.202938282 | 0.003663531 | 1.81% |
|  |  |  |  |  | A2E | 0.550902393 | 0.005198229 | 0.94% |
|  |  |  |  |  | A3E | 0.620285292 | 0.001585011 | 0.26% |
|  |  |  |  |  | A4E | 0.338421736 | 0.004815644 | 1.42% |
|  |  |  |  |  | A2F0E | 0.778439798 | 0.002917506 | 0.37% |
|  |  |  |  |  | A3F0E | 0.63462958 | 0.001630138 | 0.26% |
|  |  |  |  |  | A4F0E | 0.222188717 | 0.01768819 | 7.96% |
|  |  |  |  |  | A2FE | 0.320253499 | 0.009500396 | 2.97% |
|  |  |  |  |  | A3FE | 0.562857346 | 0.001672991 | 0.30% |
|  |  |  |  |  | A4FE | 0.098509106 | 0.012672543 | 12.86% |
|  |  |  |  |  | A2GS | 0.708558078 | 0.004788687 | 0.68% |
|  |  |  |  |  | A4GS | 0.929768661 | 0.004203507 | 0.45% |
|  |  |  |  |  | A2F0GS | 0.878160032 | 0.003146391 | 0.36% |
|  |  |  |  |  | A4F0GS | 0.913203346 | 0.006010699 | 0.66% |
|  |  |  |  |  | A2FGS | 0.473140614 | 0.009863318 | 2.08% |
|  |  |  |  |  | A2GL | 0.060243225 | 0.001343654 | 2.23% |
|  |  |  |  |  | A2F0GL | 0.070395504 | 0.001080013 | 1.53% |
|  |  |  |  |  | A2FGL | 0.04527037 | 0.001167467 | 2.58% |
|  |  |  |  |  | A2GE | 0.65036753 | 0.003976701 | 0.61% |
|  |  |  |  |  | A4GE | 0.527610845 | 0.016250197 | 3.08% |
|  |  |  |  |  | A2F0GE | 0.810274795 | 0.00208517 | 0.26% |
|  |  |  |  |  | A4F0GE | 0.410351109 | 0.006291017 | 1.53% |
|  |  |  |  |  | A2FGE | 0.439730748 | 0.010191163 | 2.32% |

**Supplemental Table S5. Main replicated associations between plasma *N*-glycan traits and clinical manifestations or clinical parameter in the discovery and validation cohorts**

| **Glycan traits** | **p-value in the discovery cohort** | **p-value in the validation cohort** | **Positive or negative association** |
| --- | --- | --- | --- |
| **Glycan traits associating with the occurance of laryngeal angioedema** | | | |
| A2G | 0.0332 | 0.0001 | positive |
| **Glycan traits associating with the occurance of gastrointestinal angioedema** | | | |
| A3L | 0.0427 | 0.0030 | negative |
| A2F0L | 0.0277 | 0.0083 | negative |
| A2F0GL | 0.0327 | 0.0095 | negative |
| **Glycan traits associating with disease severity score** | | | |
| A2G | 0.0437 | 0.0201 | positive |
| **Glycan traits associating with the level of CI-INH** | | | |
| A3F | 0.0039 | 0.0042 | positive |
| A3LF | 0.0098 | 0.0068 | positive |
| A3EF | 0.0034 | 0.0044 | positive |
| A3F0S | 0.0095 | 0.0320 | negative |
| A4GE | 0.0006 | 0.0376 | positive |

**Supplemental figures**

**Supplemental Figure S1. Boxplots of derived glycan traits changing in untreated HAE patients compared to that in healthy controls in the discovery cohort.** The boxplot for the group of HAE patients after receiving treatment is also shown. ***: p-value < 0.001, **: p-value < 0.01, *: p-value < 0.05, ns: not significant (after multiple testing correction). HAE= hereditary angioedema, HC=healthy control.


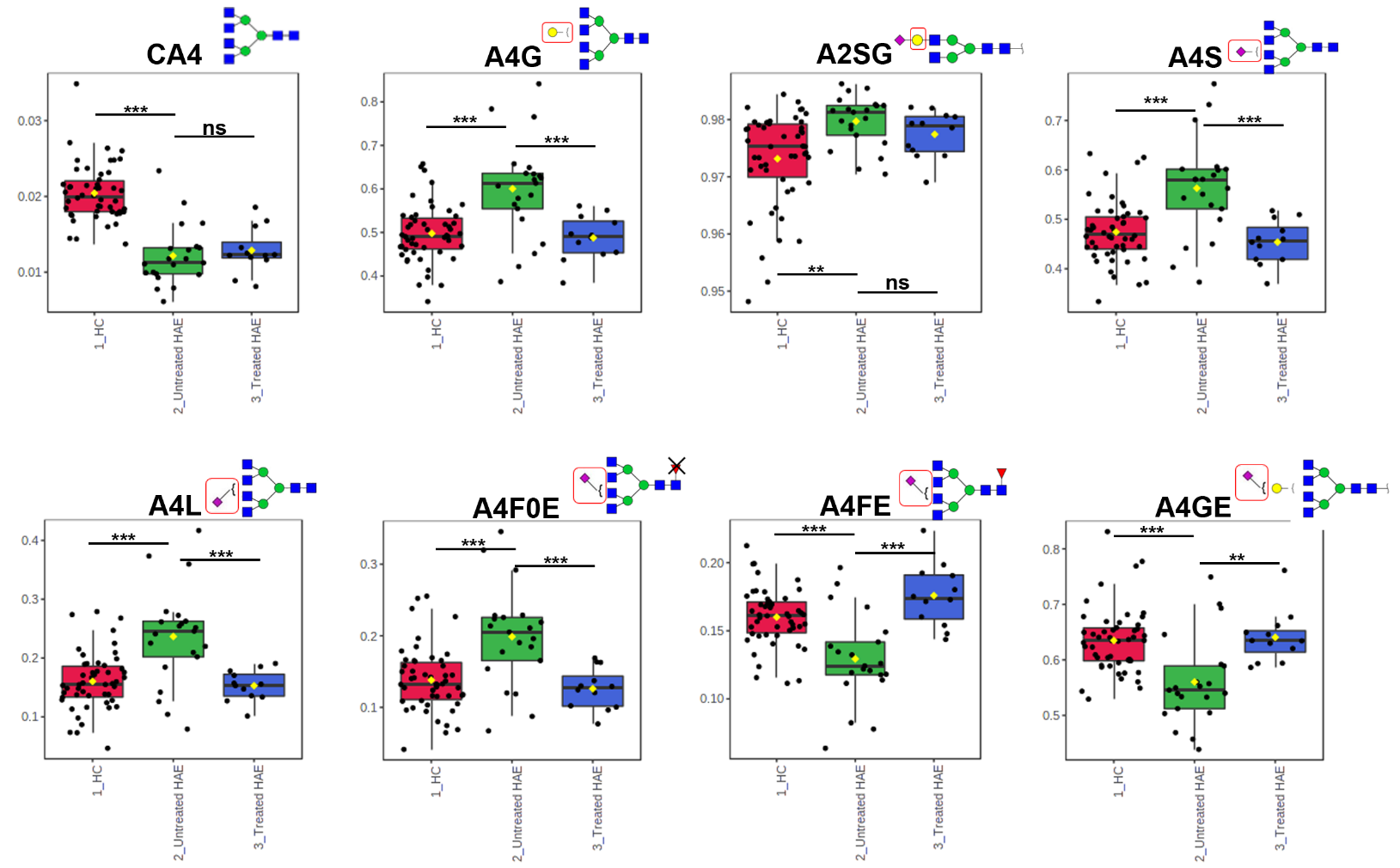


**Supplemental Figure S2. Classification models for differentiating between HAE and healthy controls based on the eight differentially expressed glycan traits in the discovery cohort.** (A) ROC curves for the top seven putative biomarker models, based on the average performance of each model across all Monte Carlo cross validation (MCCV) runs. (B) Plot of the classification accuracy of the classification models with an increased number of glycan traits. The most accurate model is marked with a red dot. (C) Plot of the classification between case and control using a single biomarker model. Due to the equilibrium of the subsampling, the classification boundary is at the center (x=0.5). Selected model: seven. (D) Plot of the most important glycan traits according to the classification models (from most to least important). HAE=hereditary angioedema, HC= healthy control.


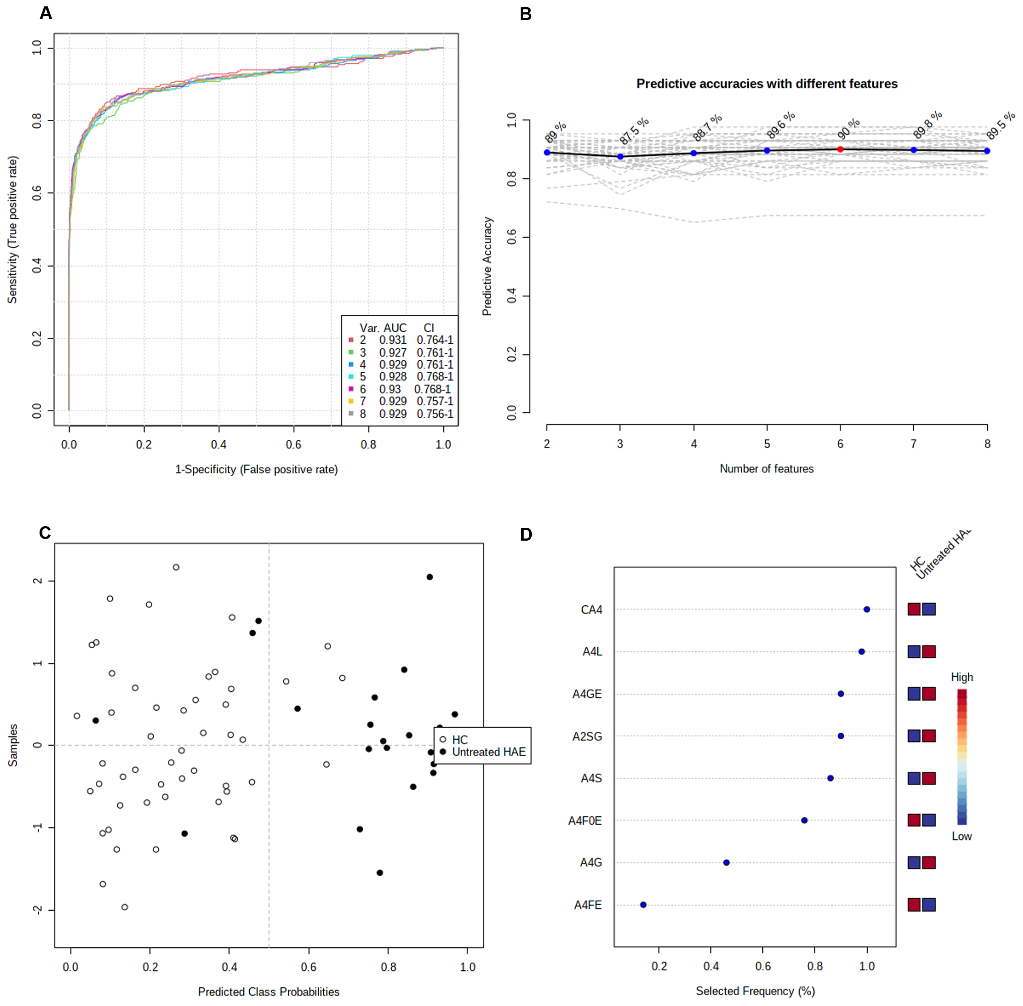


**Supplemental Figure S3. Performance of individual glycan traits in distinguishing between hereditary angioedema and healthy controls in the discovery cohort.**


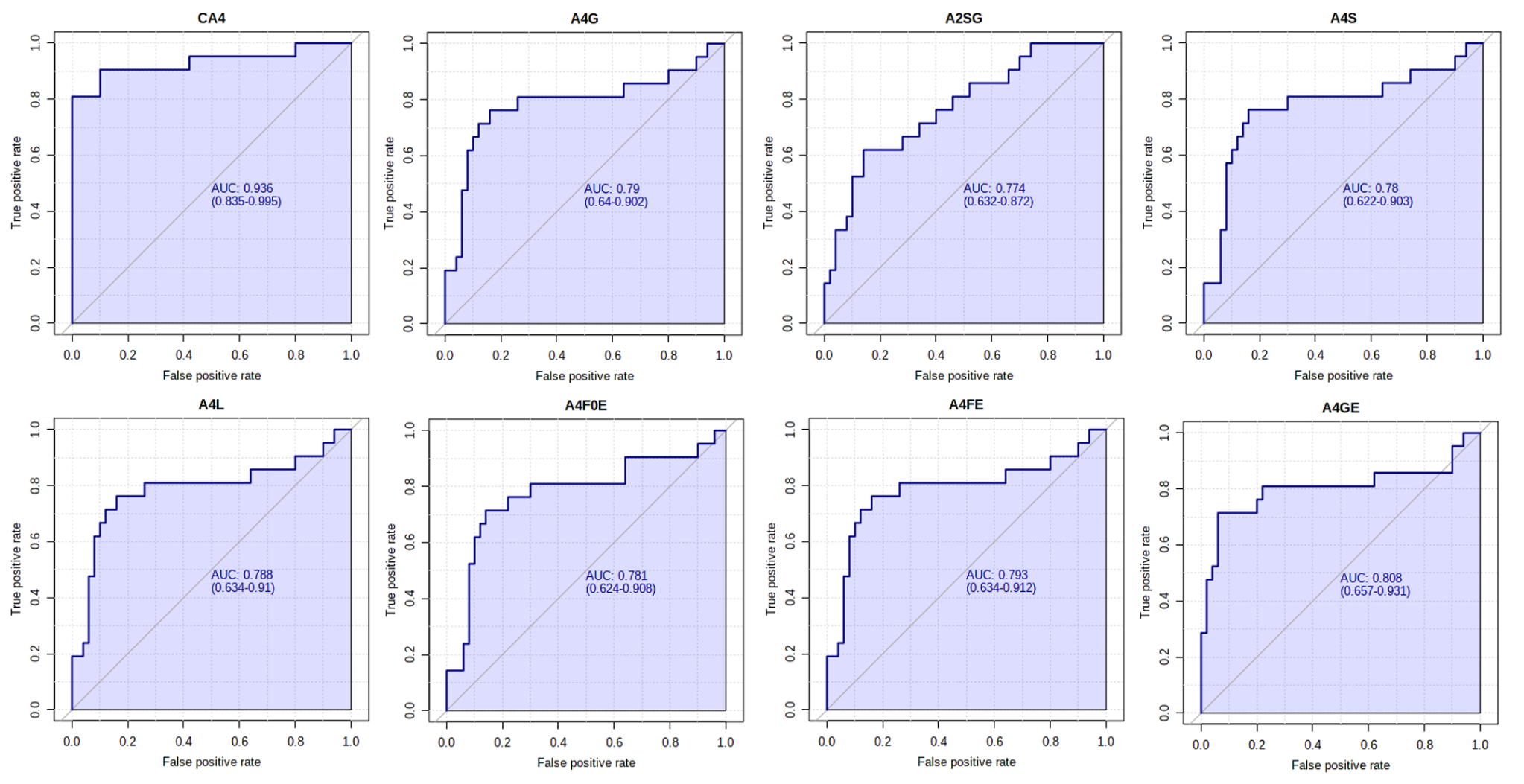


**Supplemental Figure S4. Performance of individual glycan traits in distinguishing between hereditary angioedema and healthy controls in the validation cohort.**


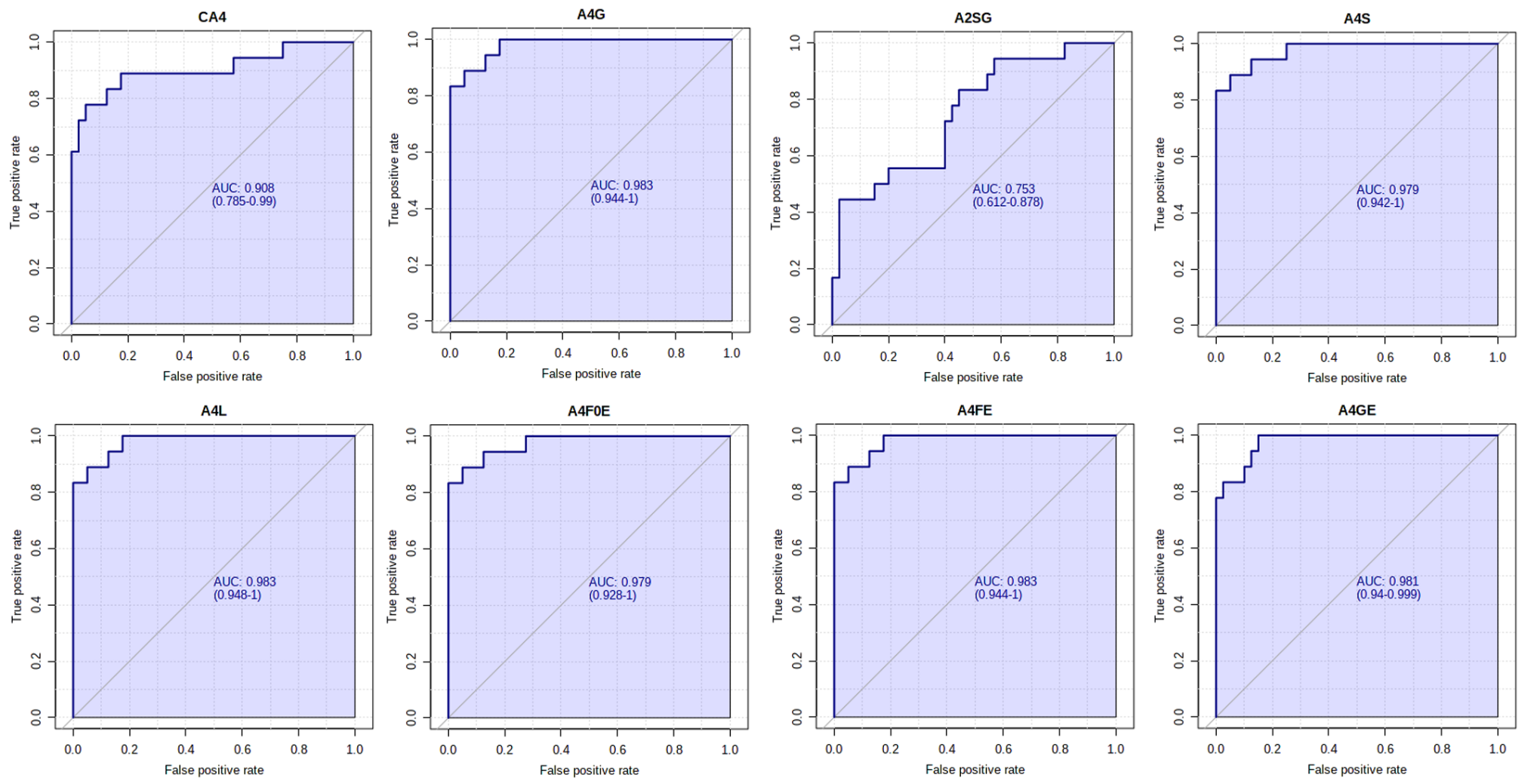


**Supplemental Figure S5. The performance of monitoring models for assessing therapeutic efficacy based on the derived glycans traits which responded to treatment (A) in the discovery cohort and (B) in the validation cohort.** ROC curves were generated for the top five biomarker models based on their average performance across all MCCV runs.


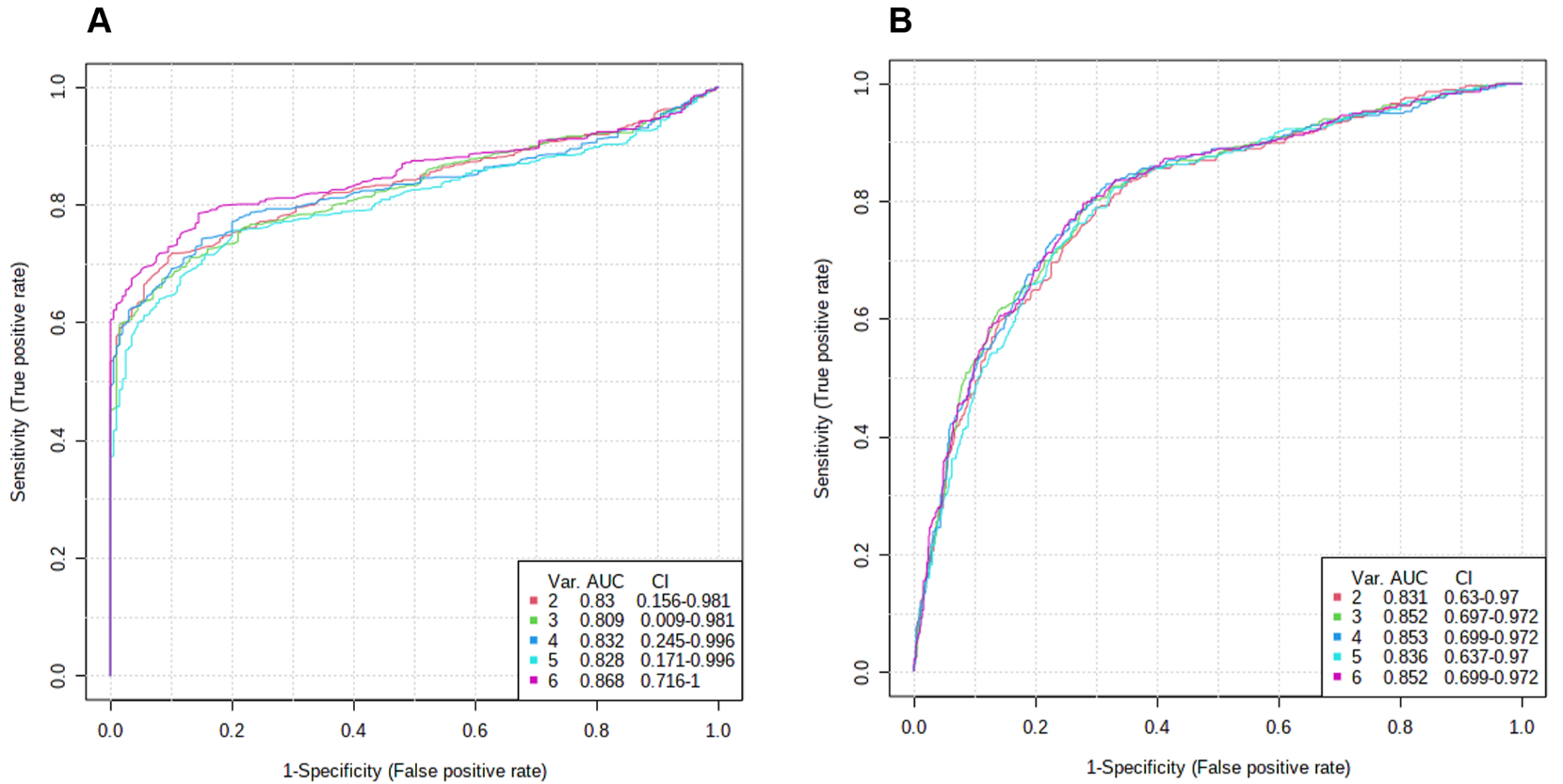


**Supplemental Figure S6. The performance of predictive models for predicting the occurrence of gastrointestinal angioedema in HAE patients based on the three replicated glycan traits with strong associations with the occurrence of gastrointestinal angioedema (A) in the discovery cohort and (B) in the validation cohort.**


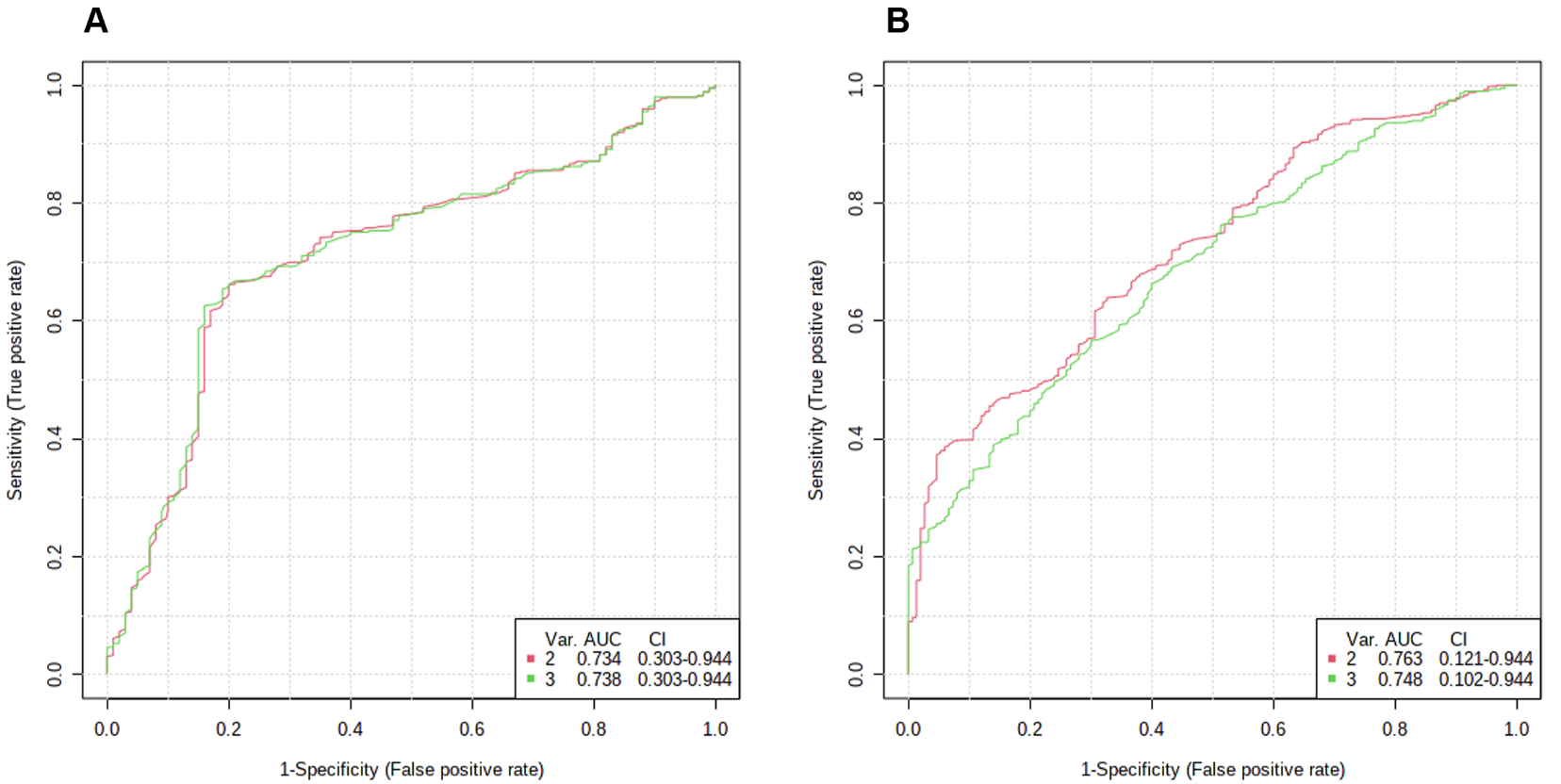

Supplement: Supplementary file 1 — Supplementary Material S1 [file CLT2-11-e12090-s001.docx]
